# Multi-faceted metagenomic analysis of spacecraft associated surfaces reveal planetary protection relevant microbial composition

**DOI:** 10.1371/journal.pone.0282428

**Published:** 2023-03-22

**Authors:** Sarah K. Highlander, Jason M. Wood, John D. Gillece, Megan Folkerts, Viacheslav Fofanov, Tara Furstenau, Nitin K. Singh, Lisa Guan, Arman Seuylemezian, James N. Benardini, David M. Engelthaler, Kasthuri Venkateswaran, Paul S. Keim

**Affiliations:** 1 Pathogen and Microbiome Division, The Translational Genomics Research Institute, Flagstaff, Arizona, United States of America; 2 Jet Propulsion Laboratory, California Institute of Technology, Biotechnology and Planetary Protection Group, Pasadena, California, United States of America; 3 Pathogen & Microbiome Institute (PMI), Northern Arizona University, Flagstaff, Arizona, United States of America; 4 School of Informatics, Computing and Cyber Systems, Northern Arizona University, Flagstaff, Arizona, United States of America; 5 Department of Biological Sciences, Northern Arizona University, Flagstaff, Arizona, United States of America; Shanghai Public Health Clinical Center, Fudan University, CHINA

## Abstract

The National Aeronautics and Space Administration (NASA) has been monitoring the microbial burden of spacecraft since the 1970’s Viking missions. Originally culture-based and then focused 16S sequencing techniques were used, but we have now applied whole metagenomic sequencing to a variety of cleanroom samples at the Jet Propulsion Lab (JPL), including the Spacecraft Assembly Facility (SAF) with the goals of taxonomic identification and for functional assignment. Our samples included facility pre-filters, cleanroom vacuum debris, and surface wipes. The taxonomic composition was carried out by three different analysis tools to contrast marker, k-mer, and true alignment approaches. Hierarchical clustering analysis of the data separated vacuum particles from other SAF DNA samples. Vacuum particle samples were the most diverse while DNA samples from the ISO (International Standards Organization) compliant facilities and the SAF were the least diverse; all three were dominated by Proteobacteria. Wipe samples had higher diversity and were predominated by Actinobacteria, including human commensals *Cutibacterium acnes* and *Corynebacterium* spp. Taxa identified by the three methods were not identical, supporting the use of multiple methods for metagenome characterization. Likewise, functional annotation was performed using multiple methods. Vacuum particles and SAF samples contained strong signals of the tricarboxylic acid cycle and of amino acid biosynthesis, suggesting that many of the identified microorganisms have the ability to grow in nutrient-limited environments. In total, 18 samples generated high quality metagenome assembled genomes (MAG), which were dominated by *Moraxella osloensis* or *Malassezia restricta*. One *M*. *osloensis* MAG was assembled into a single circular scaffold and gene annotated. This study includes a rigorous quantitative determination of microbial loads and a qualitative dissection of microbial composition. Assembly of multiple specimens led to greater confidence for the identification of particular species and their predicted functional roles.

## Introduction

One of the key goals of space exploration is the quest for extraterrestrial life; critically, it is important to prevent contamination of extraterrestrial sites with terrestrial organisms (“forward contamination”). Spacecraft must be assembled in metagenomically-monitored cleanrooms. The Jet Propulsion Laboratory (JPL) has several such cleanrooms, with various International Organization for Standardization (ISO) classifications from 5 to 8.5 (10^5^–5x10^8^ particles/m^3^). These cleanrooms are maintained with rigorous cleaning routines and high-efficiency particulate air (HEPA) or ultra-low particulate air (ULPA) air filtration systems. Depending on ISO classification, different levels of personal protective equipment are required to maintain the respective standard of cleanliness. As a result, samples obtained from these facilities are ultra-low biomass. Most of the organisms expected to be found in these environments are of human origin, although previous studies have identified the presence of oligotrophs in such facilities [1].

The National Aeronautics and Space Administration (NASA) has been monitoring the microbial bioburden of spacecraft since the 1970’s Viking missions using culture-based techniques [2] to abide by the international treaty set forth by the Committee on Space Research (COSPAR). Over the years, more comprehensive, non-culture-based techniques have been developed and tested for profiling microbial communities in the built environment [3,4]. NASA is now evaluating the feasibility of a metagenomics-based approach for the quantification, phylogenetic identification, and functional assessment of microbes on spacecraft-associated surfaces, including the cleanrooms in which they are assembled. Of particular concern are organisms resistant to decontamination methods or those with phenotypic traits that may allow survival in space environments, such as resistance to desiccation, radiation, ionization, *etc*. (Table 1).

**Table 1:** Organisms of concern to planetary protection.

Genus (species)	Phylum	Feature(s)	Reference (PMID or citation)
*Arthrobacter agilis*	Actinobacteria	psychrotroph	7547308, 27571579
*Blastococcus*	Actinobacteria	UV, radiation, desiccation resistant	26125681
*Curtobacterium flaccumfaciens*	Actinobacteria	UV resistant, Chemo-organotroph	[5]
*Geodermatophilus obscurus*	Actinobacteria	UV, radiation, desiccation resistant	26125681
*Kocuria palustris*	Actinobacteria	Chemo-organotroph, perchlorate resistant, persister	21295817
*Modestobacter spp*.	Actinobacteria	UV, radiation, desiccation resistant	26125681
*Clavibacter michiganensis*	Actinobacteria	UV resistant	[5]
*Chryseobacterium indologenes*	Bacteroidetes		
*Hymenobacter*	Bacteroidetes	Radiation resistant	29194980
*Pedobacter*	Bacteroidetes	psychrophile	
*Deinococcus radiodurans*	Deinococcus-Thermus	UV, radiation, desiccation resistant	
*Bacillus coagulans*	Firmicutes	UV resistant, spore former	[5]
*Gemmatirosa kalamazooensis*	Gemmatimonadetes	oligotroph	[6]
*Brevundimonas*	Proteobacteria	Radiation resistant	33200556
*Enhydrobacter aerosaccus*	Proteobacteria	Chemo-organotroph	
*Luteimonas*	Proteobacteria	Psychrophile Arsenic and toxin resistant	26690083
*Massilia*	Proteobacteria		
*Methylobacterium extorquens*	Proteobacteria		
*Methylobacterium populi*	Proteobacteria		
*Pantoea vagans*	Proteobacteria		
*Paraburkholderia fungorum*	Proteobacteria		
*Pseudomonas psychrotolerans*	Proteobacteria		
*Roseomonas (radiodurans)*	Proteobacteria	Radiation resistant	
*Sphingomonas*	Proteobacteria	Psychrophile Radiation resistant	29194980
*Stenotrophomonas maltophilia*	Proteobacteria	Heavy metal resistant	[7]

Two prior studies have used whole metagenome sequencing (WMGS) to evaluate microbial contamination in spacecraft assembly facilities. In one study, which included samples from the JPL Spacecraft Assembly Facility (SAF) [8], *Acinetobacter lwoffii* was dominant. The second study examined three different cleanroom facilities, including the JPL SAF [9], where again, the dominant genus during metagenome assembly was *Acinetobacter*. While culture dependent methods were not used in this study, the metagenomic approach was previously validated using focused 16S gene sequencing [8]. Pathogens and corresponding virulence genes were also found. A limitation of this study was that multiple displacement amplification was used to amplify each sample. This approach overcame technical problems with low biomass samples, but it is also known to introduce bias and contamination [10].

Here we used only unbiased shotgun metagenomics to evaluate microbial burden and composition in different cleanrooms and different types of samples collected at the JPL, developing a pipeline for continued monitoring of relevant built environments where NASA’s life-detection mission components were assembled. The assembly facilities are controlled cleanroom environments (certified ISO 5 to ISO 8.5) and were sampled using surface liquid swipes and air vacuum devices. Procedural and reagent controls were included to understand background signal. To these samples, we applied methods first to quantitate both bacterial and fungal load, and developed a two-tiered approach to generate high-quality and low-quality sequencing libraries based on biomass. We used three different classification methods to capture each sample’s most complete taxonomic profile. Likewise, we annotated the data to make functional predictions that could be important to identify unknown species representing potential high consequence space contaminants. Because of the low diversity of samples, genome assembly from the WMGS data was possible and demonstrated here for a strain identified as *Moraxella osloensis*.

## Materials and methods

### Samples and DNA extraction

The project’s experimental design was developed to comprehensively evaluate a metagenomic processing workflow and analysis pipeline for spaceflight hardware cleanroom samples (Table 2). Samples were chosen to represent 1) a range of cleanroom classifications (from ISO 5 to ISO 8.5), 2) several sample types (surface, filter, vacuum), and 3) a range of expected biomass content (from below detection limit to nanograms). Samples and their respective negative controls were sent for metagenomic analysis at various stages of the processing workflow to capture any variability and bias introduced at each critical step.

**Table 2:** Samples used in the study.

Sample Name	Sample Type	Cleanroom	Classification	Sampled Area (m^2^)	Amount Available
1–1	DNA	170–213	ISO 7	17.5	10 μL
1–2	DNA	Facility Control	NA	NA	10 μL
1–3	DNA	233–151	ISO 5	22	10 μL
1–4	DNA	Extraction Control	NA	NA	10 μL
1–5	DNA	233–151	ISO 5	NA	10 μL
1–6	DNA	Filter Control	NA	NA	10 μL
1–7	DNA	233–151	ISO 5	27	10 μL
1–8	DNA	179–121	ISO 7	NA	10 μL
1–9	DNA	103–110	ISO 8.5	5	10 μL
1–10	DNA	103–110	ISO 8.5	4	10 μL
1–11	DNA	103-102C	ISO 6	3	10 μL
1–12	DNA	233–151	ISO 5	17	10 μL
2–1	Wipe Solution	170–213	ISO 7	17.5	40 mL
2–2	Wipe Solution	233–151	ISO 5	22	40 mL
2–3	Wipe Solution	Facility Control	NA	N/A	40 mL
2–4	Wipe Solution	233–151	ISO 5	27	40 mL
2–5	Wipe Solution	103-102C	ISO 6	3	40 mL
2–6	Wipe Solution	103–110	ISO 8.5	5	40 mL
2–7	Wipe Solution	103–110	ISO 8.5	4	40 mL
2–8	Wipe Solution	103–110	ISO 8.5	4	40 mL
2–9	Wipe Solution	103–110	ISO 8.5	4	40 mL
2–10	Wipe Solution	233–151	ISO 5	17	40 mL
2–11	Wipe Solution	103-102C	ISO 6	3	40 mL
2–12	Wipe Solution	Extraction Control	NA	NA	40 mL
3–1	Filter Solution	233–151	ISO 5	NA	20 mL
3–2	Filter Solution	Filter Control	NA	NA	20 mL
3–3	Filter Solution	233–151	ISO 5	NA	30 mL
3–4	Filter Solution	233–151	ISO 5	NA	30 mL
3–5	Filter Solution	233–151	ISO 5	NA	30 mL
3–6	Filter Solution	233–151	ISO 5	NA	30 mL
3–7	Filter Solution	318–123	ISO 8	NA	30 mL
3–8	Filter Solution	306	ISO 7	NA	30 mL
3–9	Filter Solution	233–151	ISO 5	NA	30 mL
3–10	Filter Solution	Filter Control	NA	NA	30 mL
4–1	Vacuum Particle Solution	179–121	ISO 7	NA	30 mL
4–2	Vacuum Particle Solution	179–121	ISO 7	NA	30 mL
4–3	Vacuum Particle Solution	179–121	ISO 7	NA	30 mL
4–4	Vacuum Particle Solution	179–121	ISO 7	NA	30 mL
4–5	Vacuum Particle Solution	179–121	ISO 7	NA	30 mL
4–6	Vacuum Particle Solution	233–141	ISO 5	NA	30 mL
4–7	Vacuum Particle Solution	233–141	ISO 5	NA	30 mL
4–8	Vacuum Particle Solution	233–141	ISO 5	NA	30 mL
4–9	Vacuum Particle Solution	233–141	ISO 5	NA	30 mL
4–10	Vacuum Particle Solution	233–141	ISO 5	NA	30 mL
5–1	DNA	179	ISO 7	NA	10 μL
5–2	DNA	179	ISO 7	NA	10 μL
5–3	DNA	179	ISO 7	NA	10 μL
5–4	DNA	179	ISO 7	NA	10 μL
5–5	DNA	179	ISO 7	NA	10 μL
5–6	DNA	179	ISO 7	NA	10 μL
Buffer Control	Control	NA	NA	NA	40 mL
Elution Buffer	Control	NA	NA	NA	10 μL

JPL Buildings: 103, Fabrication Shop; 170, Spacecraft Fabrication Facility; 179, Spacecraft Assembly Facility (SAF); 233, Spacecraft Development Engineering Building; 306, Observational Instruments Laboratory; 318, Mobility and Robotics Technology.

NA, not applicable.

### Sample collection and processing

A total of 50 samples (41 samples and 9 controls), collected from controlled cleanroom environments (certified ISO 5 to ISO 8.5) at JPL, were sent on dry ice at -20°C to the Translational Genomics Research Institute (TGen) for DNA extraction and WMGS analysis. Samples came in the form of extracted DNA, (n = 18) and pre-extraction homogenized samples (n = 32). The samples encompassed five sample categories: 1) DNA from cleanroom facilities; 2) wipe solution from cleanroom facilities; 3) bench pre-filter solution; 4) vacuum particle solution; 5) DNA technical replicates from one cleanroom (SAF). All samples are described in detail in Table 2. All pre-extraction samples were kept cold at 4°C until processed. Following extraction, all DNA samples were frozen at -20°C.

#### Category 1: DNA samples extracted at JPL (n = 12)

A subset of the sample types described above were extracted for DNA at JPL (n = 9). All sample solutions underwent filter concentration to increase biomass concentration prior to extraction. Samples were filtered (Millipore Sigma, 50kD Amicon^®^ Ultra 15 mL Centrifugal Filters) to a final volume of 250 μL. Following the kit protocol, 200 μL of lysis buffer (Qiagen, Buffer ATL, UCP Pathogen Mini Kit) was added to each concentrated sample and incubated at 56°C for 10 min with continuous shaking at 600 rpm. Following chemical lysis, each sample was added to bead beating tubes (OPS Diagnostics, 100 μm/400 μm Acid-Washed Silica Beads) and vortexed at max speed for 10 min ± 30 sec. Final lysate was centrifuged for 2 min at 13,000 x g and 400ul were aspirated from the tubes and loaded onto a QIAcube automated DNA extraction instrument (Qiagen) and extracted following manufacturer specifications for the UCP Pathogen Mini Kit. Ten μL of DNA extract per sample were sent to TGen for sequencing and analysis. The materials such as facility wipe without active sample collection, extraction reagent, and filter were included as background controls (n = 3).

#### Category 2: Facility surface wipe samples (n = 12)

Cleanroom surface samples (n = 10) were collected from the floor, walls, and workbenches of several JPL cleanrooms used for spaceflight hardware assembly. Pre-moistened, sterile polyester wipes (Texwipe Co., TX3211 SterileWipe, ThermoFisher Scientific, UltraPure^™^ DNase/RNase-Free Distilled Water) were used for sample collection and stored in sterile 50 mL polystyrene tube (BD Falcon, 50mL Conical Tube). Wipes were transferred to individual 500 mL polystyrene storage bottles (Corning) with 100mL of dissociation buffer (10 mM Tris, 1 mM EDTA, pH 8.0, 0.05% Tween 80 [v/v]). The bottles were then sonicated for 5 min ± 15 sec at 25 kHz in an ultrasonic bath containing 0.05% Tween (v/v). For additional physical agitation, bottles were shaken at 200 rpm for 30 min ± 1 min on an orbital shaker. Since wipe solutions were expected to have highly diluted biomass, up to 2 samples from the same cleanroom and collected on the same day were combined and filter concentrated using a filter size that is small enough to retain microbial cells (Millipore Sigma, 50kD Amicon^®^ Ultra 15 mL Centrifugal Filters). 40 mL of wipe solution were sent to TGen to be used for extraction and analysis. A background control wipe solution (n = 2) followed the same protocol as all surface wipe samples, but was not exposed to the cleanroom environment, was included.

#### Category 3: Flow bench pre-filter samples (n = 10)

JPL cleanrooms contain ISO 5 flow benches that are often used for hardware assembly and 8 samples, and 2 controls were included in this category. These flow benches contain pre-filters and HEPA filters. The pre-filters are replaced at the time of recertification and collected for this study. To remove particles on the surface and within the fibers of the pre-filter, a forensic vacuum (3M, Trace Evidence Vacuum A-6510) with pre-sterilized vacuum filters (3M, Trace Evidence Vacuum Filters A-6512, 97% retention rate for 0.1-micron particles) were used. To maximize recovery, the inlet side of the flow bench pre-filters were vacuumed a total of three times. Vacuum filters were individually transferred to a polystyrene 500 ml storage bottle (Corning, Corning, NY) and 50mL of dissociation buffer (10 mM Tris, 1 mM EDTA, pH 8.0, 0.05% Tween 80 [v/v]) was added. Similar to the cleanroom wipes, bottles were sonicated for 5 min ± 15 sec at 25 kHz in a waterbath containing 0.05% Tween (v/v). Bottles were shaken at 200rpm for 30 min ± 1 min on an orbital shaker. 20 or 30mL of pre-filter solution was sent for extraction and analysis. Negative control filter solutions for each type of flow bench pre-filter (carbon or non-carbon) were obtained from new and unused flow bench pre-filters and followed the same particle removal protocol for further analysis.

#### Category 4: Cleanroom vacuum samples (n = 11)

Cleanroom vacuum dust bags (n = 10) were included in this study because they are likely to contain a high concentration of biomass while still being representative of microbes present in a cleanroom environment. These samples were collected from certified cleanroom vacuum dust bags (Nilfisk Inc., GM 30CR Cleanroom Vacuum) at the time of vacuum recertification. Dust bag particles were transferred polystyrene 500 mL storage bottles (Corning, Corning, NY) and 50mL of dissociation buffer (10 mM Tris, 1 mM EDTA, pH 8.0, 0.05% Tween 80 [v/v]) was added to each bottle. Bottles were sonicated for 5 min ± 15 sec at 25 kHz in an ultrasonic bath containing 0.05% Tween (v/v). Bottles were shaken at 200 rpm for 30 min ± 1 min on an orbital shaker. For each sample, 30 mL of dissociation solution was used for extraction and analysis. The dissociation buffer (10 mM Tris, 1 mM EDTA, pH 8.0, 0.05% Tween 80 [v/v]) was processed without any additional input and used as a background control (n = 1).

#### Category 5: Technical DNA replicate samples (n = 5)

To assess variability between technical replicates, five 10 μL aliquots from one DNA sample obtained from surface samples of the Spacecraft Assembly Facility (SAF, ISO 7) at JPL were sent to TGen. A background control containing DNA from a wipe that did not come into contact with any facility surfaces and followed the same extraction protocol was included.

### DNA samples extracted at TGen

DNA was extracted from the particulate and solution samples received from JPL (n = 32) using the MagMax Microbiome Ultra Nucleic Acid Isolation kit (ThermoFisher, #ABA423577) using the Kingfisher Flex System (Thermofisher, #5400620). Briefly, 20 mL of each homogenized solution or particulate sample was transferred to a 50 mL high-speed conical tube and centrifuged for 10 min at 13,000 x g. The supernatant was decanted and the resulting pellet was suspended in 200 μL molecular biology grade water. The suspension was transferred to a MagMax bead plate, and the manufacturer’s protocol was followed for the remainder of the extraction. DNAs were resuspended in 50 μL MagMax Elution Solution (ThermoFisher, #ABA423577).

### Bacterial and fungal quantitative analyses

The bacterial and fungal load of each sample (n = 50) was quantified using BactQuant and FungiQuant qPCR assays, respectively, as described previously [11] (Table 3 and S1 Table in S2 File https://zenodo.org/record/7041654). Since the quantity of fungal DNA in a sample is much lower than the amount of bacterial DNA, it is not as useful a predictor of whole metagenome library preparation success. FungiQuant was only performed on samples extracted at TGen to conserve sample volume on previously extracted DNA samples from JPL. The detection limits of the BactQuant and FungiQuant assays are approximately 100 16S rRNA copies/μL, and ten 18S rRNA copies/μL, respectively. Thus, samples with values close to these limits (*i*.*e*., within the same order of magnitude) are reported as having no quantifiable bacterial or fungal DNA.

**Table 3:** BactQuant and FungiQuant results for extracted DNA samples.

Sample	BactQuant	FungiQuant	Sample	BactQuant	FungiQuant
	16S copies/μL	18S copies/μL		16S copies/μL	18S copies/μL
1–1	108	ND	3–4	242	7
1–2	76	ND	3–5	241	7
1–3	71	ND	3–6	307	10
1–4	69	ND	3–7	35,097	985
1–5	58	ND	3–8	7,475	161
1–6	47	ND	3–9	235	68
1–7	46	ND	3–10	341	10
1–8	625	ND	4–1	90,587	6,498
1–9	11	ND	4–2	85,347	9,412
1–10	435	ND	4–3	70,456	7,180
1–11	24,080	ND	4–4	32,669	4,197
1–12	11	ND	4–5	83,042	8,492
2–1	120	NA	4–6	787	11
2–2	238	NA	4–7	1,168	9
2–3	291	4	4–8	961	9
2–4	197	3	4–9	1,013	23
2–5	96,788	1,127	4–10	1,116	4
2–6	1,911	251	5–1	63,772	ND
2–7	4,930	1,306	5–2	64,938	ND
2–8	3,919	1,538	5–3	65,063	ND
2–9	36,139	1,882	5–4	66,361	ND
2–10	253	3	5–5	53,616	ND
2–11	27,185	265	5–6	1289	ND
3–1	237	4	Buffer Blank	915	4.17
3–2	303	7	Extraction Blank (TGen)	735	0.54
3–3	450	55	Extraction Blank (JPL)	356	ND

NA indicates that no amplification was observed.

ND indicates that the assay was not performed.

Since the BactQuant and FungiQuant assays only account for potential bacterial and fungal DNA in a sample, respectively, additional testing was performed to determine if additional DNA, such as human or plant, might be present. A subset of the samples extracted at both TGen, and JPL was assessed using the Agilent Fragment Analyzer (Agilent Technologies) to visualize DNA content. Samples were analyzed using Agilent’s High Sensitivity Genomic DNA Kit (#DNF-488-0500), which has a detection limit of five picograms (equivalent to *cal*. 30–200 16S rRNA gene copies). Examples of two analyses are shown in S1 Fig in S1 File (https://zenodo.org/record/7041654). The results of fragment analysis agreed with BactQuant and FungiQuant data; that is, no quantifiable DNA was present in these samples (*e*.*g*., sample 3–6). Thus, the combination of qPCR and fragment analysis provided sufficient sample quality information to guide subsequent whole metagenome library preparations.

### DNA libraries and sequencing

We prepared 53 samples (consisting of 50 samples provided by JPL and one positive and two negative controls included by TGen) for WMGS using the Celero DNA-seq Kit (Tecan Genomics, #0360A-UDI). The Celero kit uses Tecan’s adapter-dimer-free technology, which makes it an ideal choice for low-biomass samples. Samples were sonicated to fragment genomic DNA, then size selection was performed to obtain a size range of approximately 600–700 bp. Libraries were quantified using KAPA’s Library Quantification Kit for Illumina (KAPA Biosystems, #KK4973), then were visualized on an Agilent Technologies Bioanalyzer to assess library size and quality. As an additional quality-control step, all samples were first sequenced using a Nano Kit v2 (2 x 150 bp) on the Illumina MiSeq platform. Although the Celero DNA-seq kit used is advertised to be dimer free, in situations where input DNA concentration is low or absent, adapter dimer has been observed. Since it is extremely difficult to completely remove this dimer *via* conventional clean-up methods, when sequenced, the dimer overwhelms larger, genomic DNA-derived fragments on the flow cell. Thus, based on the performance of individual samples on this trial sequencing run, and on the prior qPCR and fragment analysis data, only samples with appropriate quality libraries were sequenced separately on Illumina’s NextSeq platform using the 2 x 150 bp High Output Kit v2.

Twenty high-quality libraries (1–11, 2–5, 2–6, 2–7, 2–8, 2–9, 2–11, 3–7,3–8, 4–1, 4–2,4–3, 4–4, 4–5, 5–1, 5–2, 5–3, 5–4, 5–5, 5–6) were split evenly among two Illumina NextSeq High Output runs (2–150 bp). The remaining low quality libraries (1–1, 1–2, 1–3, 1–4, 1–5, 1–6, 1–7, 1–8, 1–9, 1–10, 1–12, 2–1, 2–2, 2–3, 2–4, 2–10, 3–1, 3–2, 3–3, 3–4, 3–5, 3–6, 3–9, 3–10, 4–6, 4–7, 4–8, 4–9, 4–10, and controls) were sequenced on a MiSeq (v2, 2 x 150 bp) to limit sequencing reagent waste, since the majority, if not all, of these samples were expected to fail. In addition, the MiSeq is less impacted by adapter dimer (Illumina, personal communication) so the probability of obtaining useable data was greater with this method.

### Sequencing metrics and quality control of sequence data

The paired reads for each sample were first analyzed with FastQC (https://www.bioinformatics.babraham.ac.uk/projects/fastqc/) to assess quality and adapter levels. Most of the low quantity libraries (from the MiSeq pool) contained greater than 90% adapter, as expected. However, there were a few samples (see below) that were identified as mid to high quality in the MiSeq pool that were also sequenced on the NextSeq to increase the yield for improved WMGS analysis.

Prior to read-based analysis and assembly, reads were run through KneadData (v 0.7.2) (http://huttenhower.sph.harvard.edu/kneaddata) to remove adapters, low quality reads and human reads. KneadData incorporates FastQC, Trimmomatic [12], and BMTagger (ftp://ftp.ncbi.nlm.nih.gov/pub/agarwala/bmtagger/) for mapping and filtering using the latest assembly of the human genome. The results from KneadData for each sample are shown in S2 Table in S2 File (https://zenodo.org/record/7041654). Note that most of the 20 samples that we selected for NextSeq sequencing (in black) performed well, although samples 5–6, 1–11, and 2–7 had significant numbers of reads that were dropped post-filter (87.7%, 35.0% and 29.6%, respectively). For the samples sequenced on the NextSeq, human filtering was very efficient, with a few exceptions, notably 2–5, where 9.1% human reads remained (an enrichment from 0.6%, probably due to loss of poor-quality reads) and sample 3–7 where 10% of reads remained from a pre-filter high of 78.4%. Examination of the MiSeq data revealed four samples (i.e., 1–8, 1–10, 2–1 and 4–7) that could be promoted to NextSeq sequencing to gather additional data.

### Sequencing depth metrics

**Fig 1:**
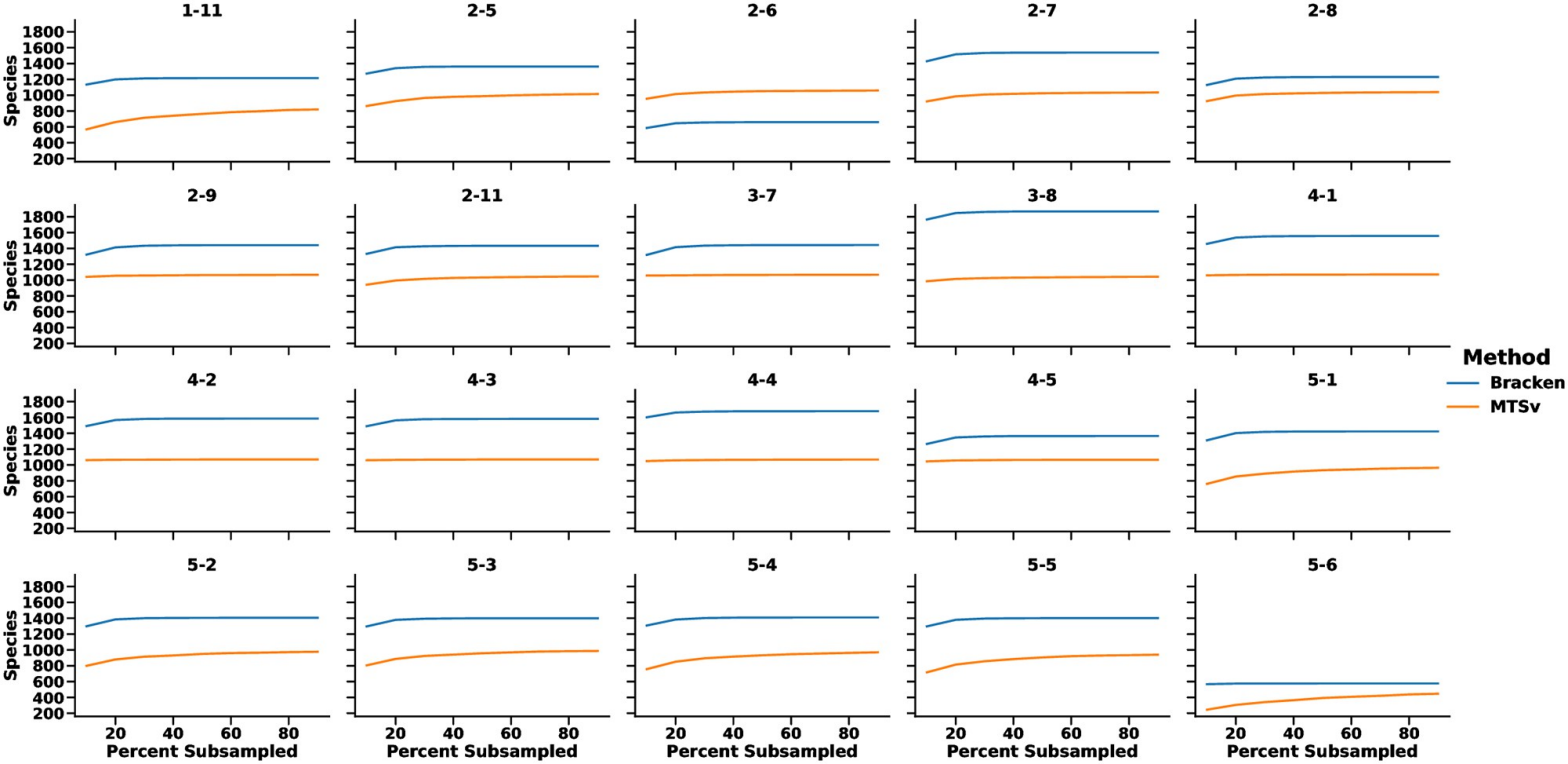
The sequencing effort was sufficient to capture most species from each sample. The plots were generated by randomly subsampling 10% to 90% of reads from each dataset and estimating the number of species identified using MTSv and Bracken. As the number of subsampled reads approach 100%, the number of species identified leveled off which suggests that all but the rarest species were captured. Bracken identified more species because it reallocates genus-level assignments to the species level. For MTSv, the species count included only species with reads that aligned uniquely at the species level. The values are the average for 10 replicates for each subsample size. The results shown are from the 20 high-quality libraries sequenced using Illumina NextSeq High Output runs.

To assess whether sufficient amounts of sequence data were generated for accurate and sensitive taxonomic characterization for the 20 high-quality libraries, we performed a series of subsampling experiments to assess the rate of species accumulation (Fig 1), similar to a rarefaction curve. The goal was to determine whether additional sequencing was needed and/or would provide substantial improvements in the number of candidate species detected for each of the samples.

The sequence reads generated for each of the high-quality libraries were subsampled (10X times) at 10% intervals from 10% to 90% of total reads for a total of 20 X 10 X 9 = 1,800 simulations. Each subsampled read set was processed via MTSv (v 1.0.0) to identify candidate species. In all cases, the number of candidate species plateaued well before the actual number of reads (100%) was achieved, suggesting that additional sequencing would not detect additional (sufficiently common) species in the samples. This also provides a prediction of the complexity of a sample. For example, the category 5 samples are predicted to have low diversity.

### Sample classification by MTSv

All sequence reads were segmented into non-overlapping 50-mer fragments (with read pairing information ignored) prior to MTSv analyses (Table 4). The resulting segments were taxonomically classified to the genus and species levels using the MTSv pipeline. The MTSv pipeline (https://github.com/FofanovLab/MTSv) uses a local copy of the GenBank database (accessed 06/15/2018) as the reference. Briefly, the flat files of the entire NCBI GenBank were downloaded; all sequences without a valid (species level or below) taxonomic classification or labeled as “environmental” samples were discarded; all sub-species (strain-level and below) taxonomic classifications were collated into their parent species classification. The resulting sequences were then used to build a custom FM-Index for fast taxonomic classification of high throughput sequencing reads. The pipeline performed a full alignment with up to three mismatches tolerated against all taxonomically classified sequences. Only the sequences that aligned uniquely to a taxonomic unit (species or genus levels) within the database were used for taxonomic classification. In this fashion, only the sequence fragments that unambiguously supported the presence of an organism were used. This significantly reduced the false positive rate associated with metagenomic characterization of complex samples.

**Table 4:** MTSv sequence fragments and alignment characteristics.

Sample ID	Total queries	Total Unique Queries	Total Hits	Unique Hits	Total Signature Hits	Unique Signature Hits
**1–11**	103,754,754	9,150,722	23,028,931	629,428	7,799,693	190,187
**2–5**	100,900,320	25,796,591	11,490,776	2,186,679	4,571,055	885,072
**2–6**	280,642,620	58,865,028	135,980,848	24,432,234	68,585,206	13,215,939
**2–7**	110,687,178	17,275,745	45,510,048	5,315,222	21,020,601	2,443,712
**2–8**	234,167,724	30,049,691	81,221,576	7,733,769	39,502,304	4,354,788
**2–9**	250,441,860	77,651,127	59,140,285	17,723,669	29,093,105	9,449,235
**2–11**	191,972,508	48,185,099	35,838,610	7,813,663	14,633,750	3,595,596
**3–8**	135,504,102	21,303,958	41,475,681	4,932,807	18,941,013	2,744,404
**4–1**	154,931,496	113,392,306	16,422,050	10,908,297	8,612,351	5,822,948
**4–2**	279,064,938	149,863,804	29,134,977	14,102,338	14,831,677	7,500,856
**4–3**	335,025,570	132,221,207	32,303,899	11,079,516	16,559,477	5,741,737
**4–4**	167,550,696	61,942,411	24,528,139	6,199,648	4,498,553	1,693,693
**4–5**	196,364,718	55,798,547	28,132,608	5,133,003	11,665,278	2,637,486
**5–1**	183,044,640	17,854,314	26,174,865	1,101,514	8,888,183	466,272
**5–2**	224,050,866	20,756,537	26,612,583	1,231,554	9,168,072	505,716
**5–3**	237,926,214	22,987,238	25,873,610	1,227,309	9,760,505	506,774
**5–4**	175,980,252	15,694,738	29,934,496	1,055,020	13,962,033	431,344
**5–5**	144,708,342	13,333,016	28,529,159	964,106	4,563,975	389,819
**5–6**	42,147,612	1,724,231	25,381,052	434,120	1,871,807	165,532

For each taxonomic unit within GenBank, MTSv provides the number of ***total hits***, the number of ***unique hits*** (i.e., unique 50-mer sequence), ***total signature hits*** (reads aligning to one and only one species), and the ***total unique signature hits***. The candidates were then ranked (high to low) in terms of ***total signature hits*** (unique in terms of sequence, reads aligning to one and only one species). This allows MTSv to avoid issues associated with spurious genomic similarity between organisms when assessing taxonomic composition of each sample. Thus, top ranked candidate taxa are both present in high proportion in the sample and identified with the highest confidence.

For each candidate taxon identified as part of the initial run of the MTSv pipeline, we performed a proportion test to provide a confidence score for the presence of the taxon. Briefly, assume that ${\hat{SR}}_{T_{i}}$ is the proportion of signature to total hits for a taxon *T*_*i*_, *where i* = 1,…,# *of candidate taxa*, whereas ${SR}_{T_{i}}$ is the true proportion of signature to total hits for this taxon (estimated from the reference sequences of the taxon). Assuming a Null Hypothesis H_0_: ${\hat{SR}}_{T_{i}}={SR}_{T_{i}}$, and an Alternative Hypothesis of H_A_: ${\hat{SR}}_{T_{i}}\neq \;{SR}_{T_{i}}$, we can then perform the proportion test to assign a likelihood that the observed proportion of signature reads conforms to expectations. The proportion test involves computing the test statistic $z=\frac{{\hat{SR}}_{T_{i}}-\;{SR}_{T_{i}}}{\sqrt{\frac{\;{SR}_{T_{i}}\left(1-{SR}_{T_{i}}\right)}{n}}}$, where *n* is the total number of trials (100,000 in our example above). With this approach, for each of the candidate taxa, we computed a *p*-value to assess our confidence in the call (*i*.*e*. our confidence in observing the signature reads we have, under the assumption that the organism was actually there). In addition to the *p*-value, we also computed the Cohen’s H statistic to measure the distance between the two proportions, as well as the actual ratio of observed versus expected proportion ratio = ${\hat{SR}}_{T_{i}}/{SR}_{T_{i}}$.

Candidate taxa with at least 300 unique signature hits, a Cohen’s H value of < 0.4, and a proportion ratio < 2.5 were considered to have very high confidence. Candidates with at least 300 unique signature hits, Cohen’s H value of < 0.5, and proportion ratio < 2.5 were considered to have high confidence. Candidates with at least 300 unique signature hits and a proportion ratio < 4 were considered to have medium confidence. Candidates not fitting these criteria were considered to have low confidence and were discarded from summary and analysis. The raw data, including ratios, and confidence determinations are available in the S3–S6 Tables in S2 File (https://zenodo.org/record/7041654).

### Taxonomic classification using Bracken and MetaPhlAn2

In addition to MTSv, two additional tools were used to taxonomically classify the reads in the high-quality samples. The first, Bracken (Bayesian Reestimation of Abundance after Classification with Kraken) [13,14] is a k-mer-based classifier that includes estimations of species abundance in a sample. Bracken was run using default parameters. MetaPhlAn2 [15] is a tool that uses a marker gene database to identify bacteria, archaea, eukaryotes, and viruses in metagenomic sequencing samples. The current database represents about 13,500 bacterial and archaeal strains, 3,500 viral, and 110 eukaryote isolates. MetaPhlAn 2.0 was run using the version 2.0 database with default parameters. Heatmaps were constructed using hclust2 (https://github.com/SegataLab/hclust2).

### Read-based functional annotation

Metabolic capacity was predicted using two methods. The first was using HUMAnN2 [16], which uses the marker species information from MetaPhlAn2 to align reads to species pangenomes, then performs a translated search of unclassified reads using a comprehensive non-redundant protein database (UniRef50) [17] followed by normalization to reads per kilobase (RPK) units. We also performed a KEGG-based functional analysis using the same segmented 50-mer fragments used in the MTSv analysis. Based on the MTSv metagenomic analysis, an estimate of the likely translation space was used to assemble a library of 6,741 functions using the KEGG Orthology (KO). Briefly, a total of 117 bacterial species with annotations in KEGG, each containing between 677 and 3,868 annotated functions, were identified. The union of these yielded 6,741 unique functions (with unique KO numbers), with many functions supported by multiple candidate bacterial taxa. The 50-mers were mapped against these 6,741 functions. Because a single sequence fragment can (and often does) align to multiple KO numbers, the signature space annotation was carried out for each of the KO number bins. Using this approach, only the reads that unambiguously supported a single function were used in the functional analysis. Reads were reported as RPK per million reads (RPKM).

### Assembly and annotation

High-quality metagenomic assembled genomes (MAG) were produced using IDBA-UD [18] using the option—pre_correction—min_contig 500. Assembly statistics were generated using assembly-stats (https://github.com/sanger-pathogens/assembly-stats). The *M*. *osloensis* MAG was assembled by recruiting reads to the *M*. *osloensis* strain MOXF1 assembly (https://www.ncbi.nlm.nih.gov/nuccore/CP040257.1). We then assembled the reads with SPAdes v. 3.14.0 with the non-default option—careful to reduce the number of mismatches and indels [19]. Annotation was performed using Prokka v 1.13 [20] using the reference genome with the options prokka—proteins Moraxella_osloensis_strain-MOXF1.gb—prefix M-osloensis-Assembly contigs.fasta—mincontiglen 200—rawproduct.

### Taxonomic assignment of MetaGenomic assemblies

Taxonomic classification was done using a local copy of NCBI’s nt database [21] downloaded on 12/07/2021. Scaffolds were aligned to the database using BLAST+ 2.12.0 software package with default parameters and “-max_target_seqs 5” flag enabled. The top hit for each scaffold was selected on the basis of NCBI’s bit score, provided that alignment covered at last 25% of the original query with at least 80% sequence identity and an e-value of 1*10^−7^ or lower. Taxonomic classification of top alignments was done using taxonomizr 0.7.1 (https://github.com/sherrillmix/taxonomizr) software package and a local version NCBI’s accession2taxid and taxdump databases downloaded on 12/07/2021.

## Results

We employed three separate established metagenomic analysis pipelines (MTSv, MetaphlAn2, and Bracken) that are based upon different methods (true alignment-based, marker-based, k-mer-based, respectively) the assign microbial content and predict function withing various low microbial burden environmental samples collected in the JPL space craft manufacturing facilities. These results are discussed individually and collectively below and put into context with current understanding of the risks of microbial flora contamination in space exploration.

### MTSv analysis

**Fig 2:**
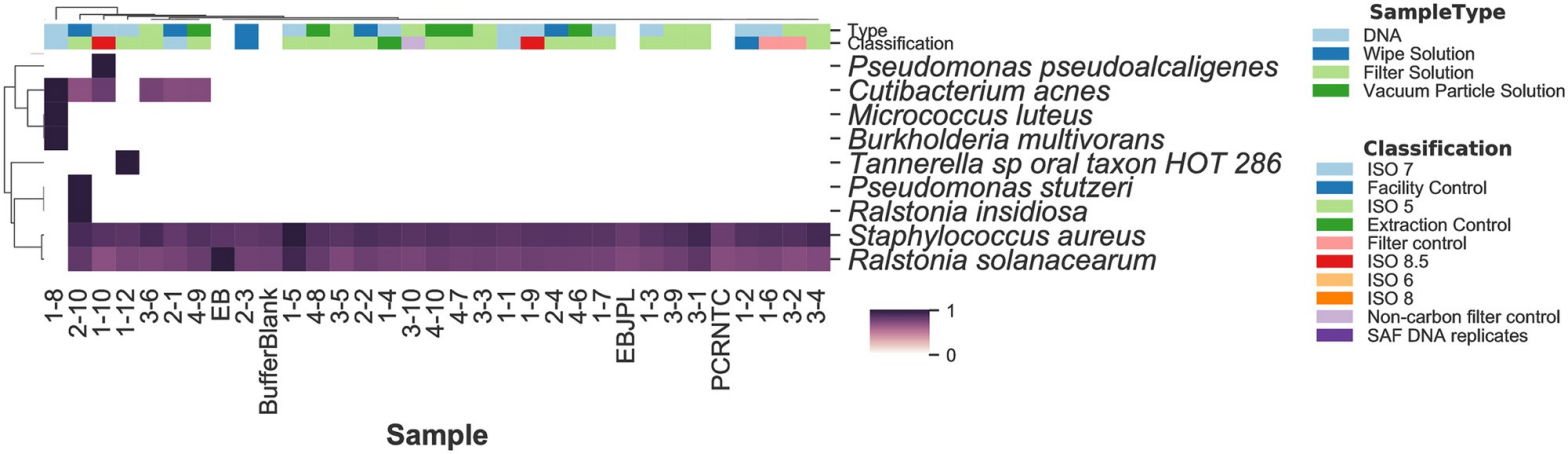
Few organisms were detected in the samples with low amounts of input DNA, and these were likely contaminants that were also found in controls and blank buffer samples. *Staphylococcus aureus and Ralstonia solanacearum* were identified in all samples including the PCR no template control (PCRNTC), the elution buffer (EBJPL) and buffer blanks. Seven additional species were found among 7 of the samples. The heat map shows the hierarchical clustering of all species-level bacterial candidate taxa with medium confidence or better (i.e., 300+ signature reads and the ratio ${\hat{SR}}_{T_{i}}/{SR}_{T_{i}}<\;4.0$) detected using MTSv. Sample type and description grouping are shown on the x-axis. The heat map colors indicate the number of signature hits assigned to each species (the values were converted to a long scale for better visualization). The values have been normalized by row to show the relative difference in species hits between samples.

As expected, when there were low amounts of input DNA the sequencing libraries were of low quality and very few organisms were detected. As shown in the Table 4 MTSv alignment metrics, this included samples:1–1, 1–2, 1–3, 1–4, 1–5, 1–6, 1–7, 1–8, 1–9, 1–10, 1–12, 2–1, 2–2, 2–3, 2–4, 2–10, 3–1, 3–2, 3–3, 3–4, 3–5, 3–6, 3–9, 3–10, 4–6, 4–7, 4–8, 4–9, 4–10, and controls (not shown). These samples contained trace amounts of *Staphylococcus aureus* and *Ralstonia solanacearum*, which are probably low-level contaminants, since they were also detected in the controls and the buffer blank samples (Fig 2). Many of these samples also contained significant human DNA (as per the MTSv pipeline, human DNA was not filtered for the MTSv analysis). Samples 1–8 and 2–10 had the largest number of non-contaminant candidate taxa, including *Cutibacterium* (formerly *Propionibacterium) acnes*, *Micrococcus luteus* and *Pseudomonas stutzeri*. Samples 1–10, 1–12, 2–1, 3–6, and 4–9 also had a few taxa, but the remaining samples had none.

**Fig 3:**
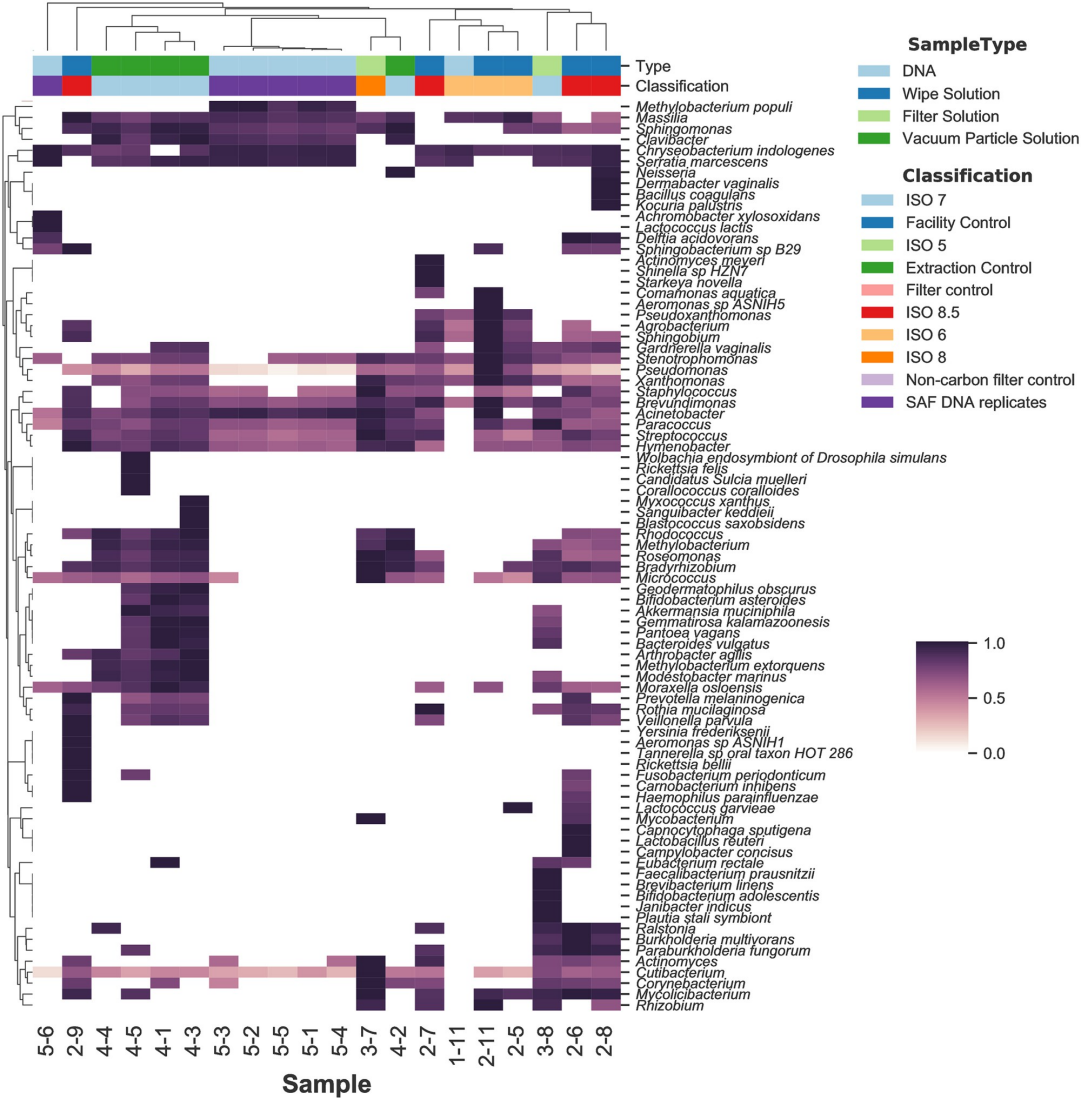
Substantial taxonomic diversity was observed among the high-quality libraries and there is some clustering of sample types. The heat map shows the hierarchical clustering of species- and genus-level bacterial candidate taxa with medium confidence or better (i.e., 300+ signature reads and the ratio ${\hat{SR}}_{T_{i}}/{SR}_{T_{i}}<\;4.0$) detected using MTSv. Sample type and description grouping are shown on the x-axis. The heat map colors indicate the number of signature hits assigned to each species (the values were converted to a long scale for better visualization). The values have been normalized by row to show the relative difference in species hits between samples.

In contrast, there was substantial taxonomic diversity observed in the high-quality libraries resulting from higher input samples. Taxonomic classification of samples was carried out at the species level (Fig 2 and S3 and S4 Tables in S2 File https://zenodo.org/record/7041654), the genus level (S5 Table in S2 File https://zenodo.org/record/7041654), and a combination of species and genus calls when confident species calls were not possible (Fig 3 and S6 Table in S2 File https://zenodo.org/record/7041654). The vacuum samples from SAF (ISO 7 samples, 4–1, 4–3, 4–4, and 4–5) formed the tightest cluster *(i*.*e*., having the least number of differences in taxonomic composition), and were dominated by *M*. *osloensis* and several organisms of concern for planetary protection. All samples contained *Methylobacterium* and some contained a combination of *Blastococcus saxobsidens*, *Modestobacter marinus*, and *Geodermatophilus obscurus*. These three organisms are members of the family Geodermatophilaceae and were originally isolated from harsh environments [22]. They are able to resist UV light, ionizing radiation, heavy metals, desiccation, starvation and oxidative stress [22,23]. Other organisms of concern in this cluster are *Sphingomonas* (a psychrophile) and *Roseomonas*, one species of which (*R*. *radiodurans)*, is radiation resistant [24]. The SAF DNA replicates (category 5) formed another tight, yet less taxonomically diverse, cluster dominated by *Methylobacterium populi*, *Massilia* spp., *Sphingomonas* spp., *Clavibacter* spp., *Serratia marcescens* and the organo-oligotroph *Chryseobacterium indologenes*. They also had a high relative abundance of *Acinetobacter* species. The similar profiles were expected since these were replicates. A notable exception and outlier was sample 5–6, which had no PCR detectable 16S or ITS sequences compared to all the other JPL samples. The wipe solution samples (category 2) did not form a cluster and had few consistently dominant organisms; these included *Acinetobacter*, *Cutibacterium*, *M*. *osloensis* and *Staphylococcus*. The category 3 samples (filters; n = 2) that were sequenced had profiles similar to most of the category 2 samples (wipes).

### Taxonomic classification using MetaPhlAn2 and Bracken

**Fig 4:**
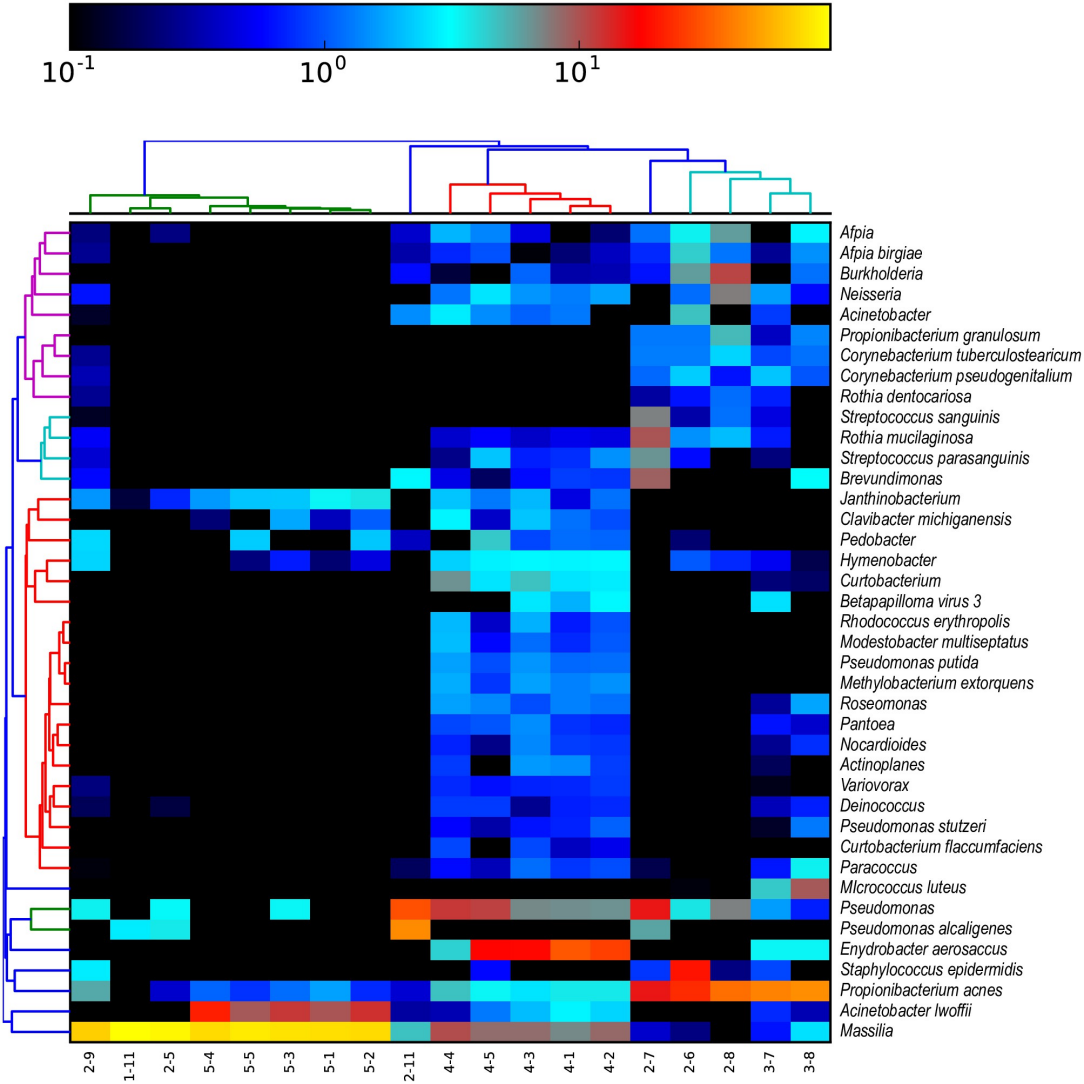
Heat map and hierarchical clustering of the top 40 taxa mapped by MetaPhlAn2. Sample category and number is shown on the x-axis. Whole metagenome sequences from each sample were characterized by MetaPhlAn2 and merged into a relative abundance table. A heatmap with hierarchical clustering of the top 40 taxa was generated to illustrate similarity.

**Fig 5:**
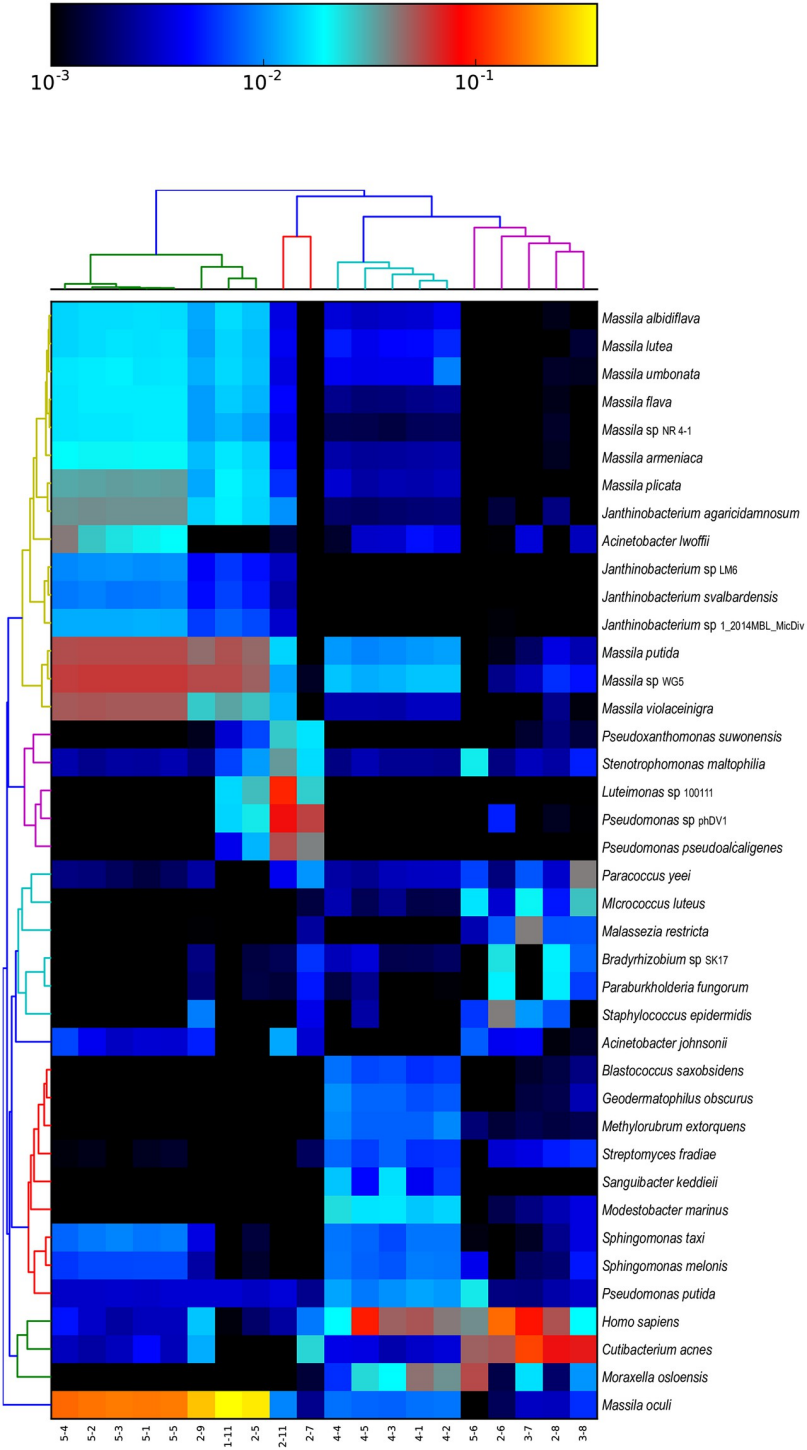
Heat map and hierarchical clustering of the top 40 taxa mapped by Bracken. Sample category and number is shown on the x-axis. Taxonomic assignments from whole metagenome sequences from each sample were assigned by Kraken2 and abundances were estimated using Bracken. The results for each sample were merged into a relative abundance table. A heatmap with hierarchical clustering of the top 40 taxa was generated to illustrate similarity.

Metagenomic reads from the high-quality libraries were also analyzed using MetaPhlAn2 [15] and Bracken [13] to identify taxa and calculate their relative abundances. Heatmaps were generated to visualize the 40 most abundant organisms assigned by each tool and to visualize clustering by sample (Figs 4 and 5). The complete data tables are available in the S7 and S8 Tables in S2 File (https://zenodo.org/record/7041654). As seen in the MTSv analysis, samples from categories 4 (vacuum particle solution samples) and 5 (DNA replicates) formed distinct clusters using both tools. As mentioned above, sample 5–6 was unlike any of the other samples and did not have any reads that could be classified by MetaPhlAn2, so it was not carried forward in that analysis. Category 5 had a high abundance of taxa in the Oxalobacteraceae family, specifically *Massilia* spp. and *Janthinobacterium* spp.; *A*. *lwoffii* and *P*. *acnes* was also present. As observed in the MTSv analysis, vacuum debris samples had a high abundance of the chemo-organotroph, *Enhydrobacter aerosaccus*, *Massilia* and presence of *A*. *lwoffii* and *P*. *acnes*. These samples also contained the desiccation and radiation resistant species identified by MTSv (Fig 5), including *B*. *saxobsidens*, *G*. *obscurus* and *M*. *marinus*. MetaPhlAn2 identified the genus *Deinococcus*; Bracken identified the spore-former *Streptomyces fradiae*. Vacuum debris samples also contained *Pseudomonas* spp., including *P*. *putida*, and the heavy metal resistant species *Stenotrophomonas maltophilia* [7]. Three wipe samples clustered (2–6, 2–7, 2–8) with the HEPA filter samples (3–7 and 3–8) and had a high abundance of *C*. *acnes*, *Pseudomonas*, and several species of *Corynebacterium* and *Sphingomonas* (Figs 4 and 5). They also had *Massilia* spp. and the presence of the fungus *Malassezia restricta*. Planetary Protection relevant taxa, mentioned above, were also present at low relative abundance. Overall, vacuum debris (category 3) samples appeared to be the most diverse and were composed mostly of members of the Actinobacteria and Proteobacteria phyla, including many hardy environmental organisms. The wipe (category 2) and ULPA filter (category 4) samples had less diversity and were predominated by the Actinobacteria phylum, including human commensal taxa. Categories 1 (one sample) and 5 were the least diverse and also dominated by Proteobacteria of both human and environmental origin.

### Metagenomic analysis of sample composition—Cross approach comparison

**Fig 6:**
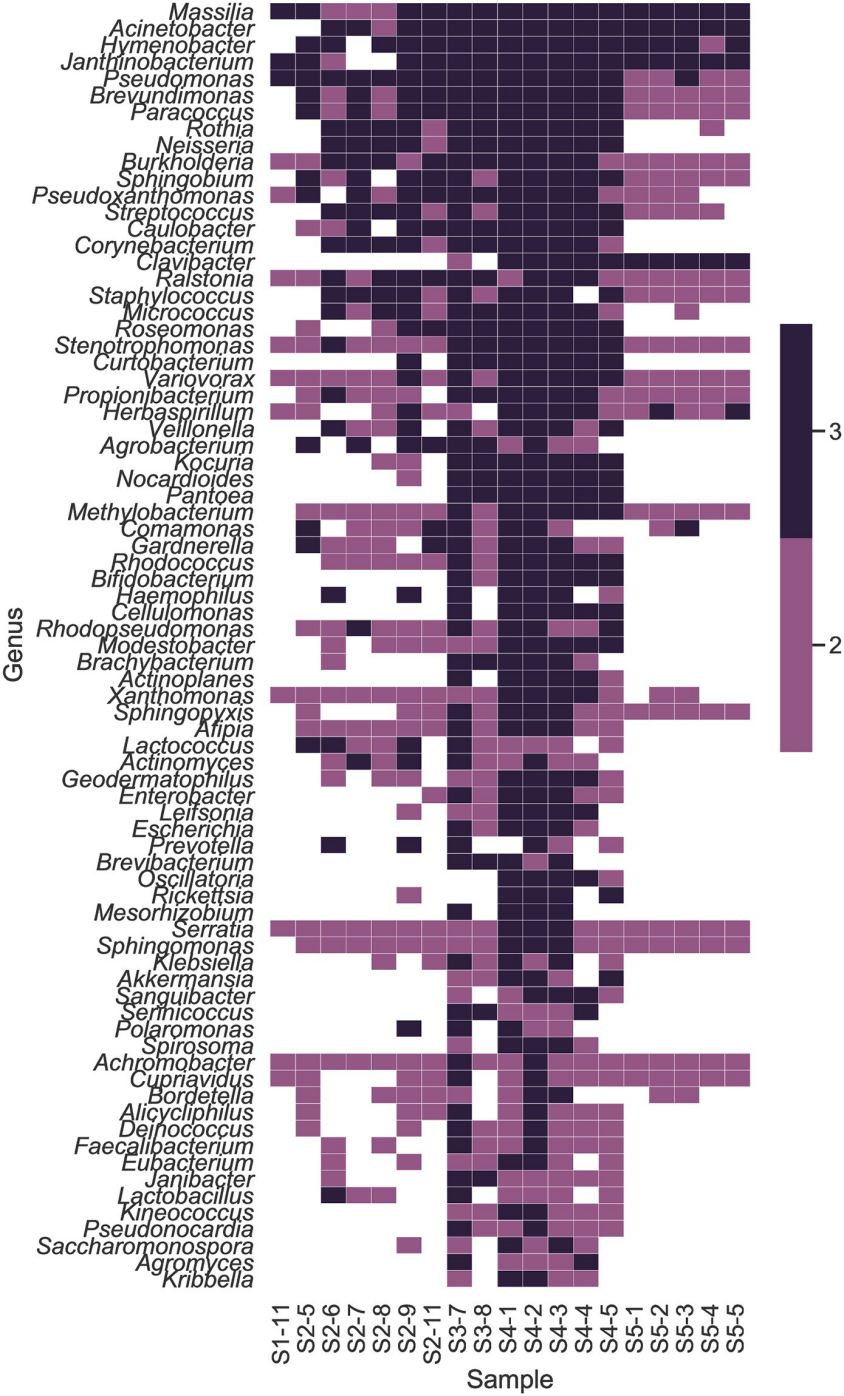
Heat map of genus-level overlap among three classification methods. The heatmap shows the number of methods (MTSv, Bracken, and MetaPhlAn2) that agree with each call at the genus level. Shown are 78 genera identified by all three methods in at least two samples. A total of 111 unique genera were identified by all three methods, 383 unique genera were identified in at least two methods, and 1,081 unique genera were identified in at least one method, across all samples. When only two methods agreed it was usually MTSv and Bracken, MetaPhlAn2 missed 93% of calls made by the other two tools.

**Fig 7:**
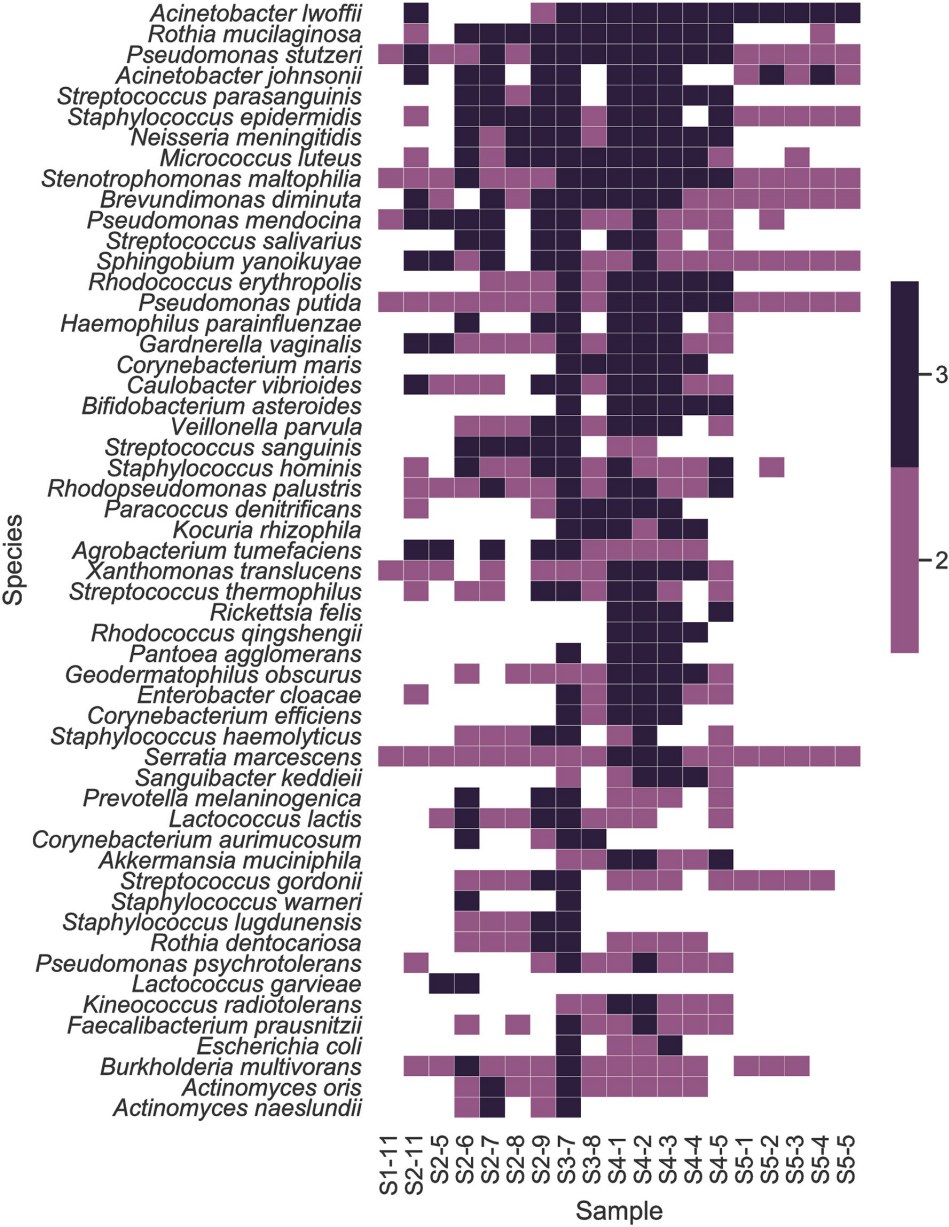
Heat map of species-level overlap among three classification methods. The heatmap shows the number of methods (MTSv, Bracken, and MetaPhlAn2) that agree with each call at the species level. Shown are the 54 species identified by all three methods in at least two samples. A total of 317 unique species were identified by all three methods,3,698 unique species were identified in at least two methods, and 26,542 unique species were identified by at least one of the methods. When only two methods agreed it was usually MTSv and Bracken, MetaPhlAn2 missed 96% of the total species-level calls.

There was considerable overlap of the genus-level calls between the three classification approaches used (Fig 6 and S9 Table in S2 File https://zenodo.org/record/7041654). Of the total 10,599 genus-level calls across all samples, 505 (5%) were made by all three methods, 1,767 (16.7%) were made by two of the methods and 8,327 (78.6%) were made by only one of the methods. When calls were made by two of the methods, MetaPhlAn2 was the method that more often failed to make a matching call (1646/1767, 93.2%). This is not surprising, since MetaPhlAn2 uses a marker gene database to map reads so it is most prone to sensitivity issues (with a tradeoff of increased specificity when the marker gene is present in the sequencing read set). Lower overlaps but similar patterns were observed when comparing species level calls for the three methods (Fig 7 and S10 Table in S2 File https://zenodo.org/record/7041654). Of the 30,557 species level calls across all samples, 317 (1%) were called by all three methods, and 3,698 were called by two methods. Again, MetaPhlAn2 was lowest with only 1,099 out of 30,557 calls. While abundance may play a role for rare taxa, the methods’ intrinsic identification differences are more important. This emphasizes the utility of using multiple methods for taxonomic composition and highlights the importance of not relying on one method or database.

### Functional analysis of samples using HUMAnN2

**Fig 8:**
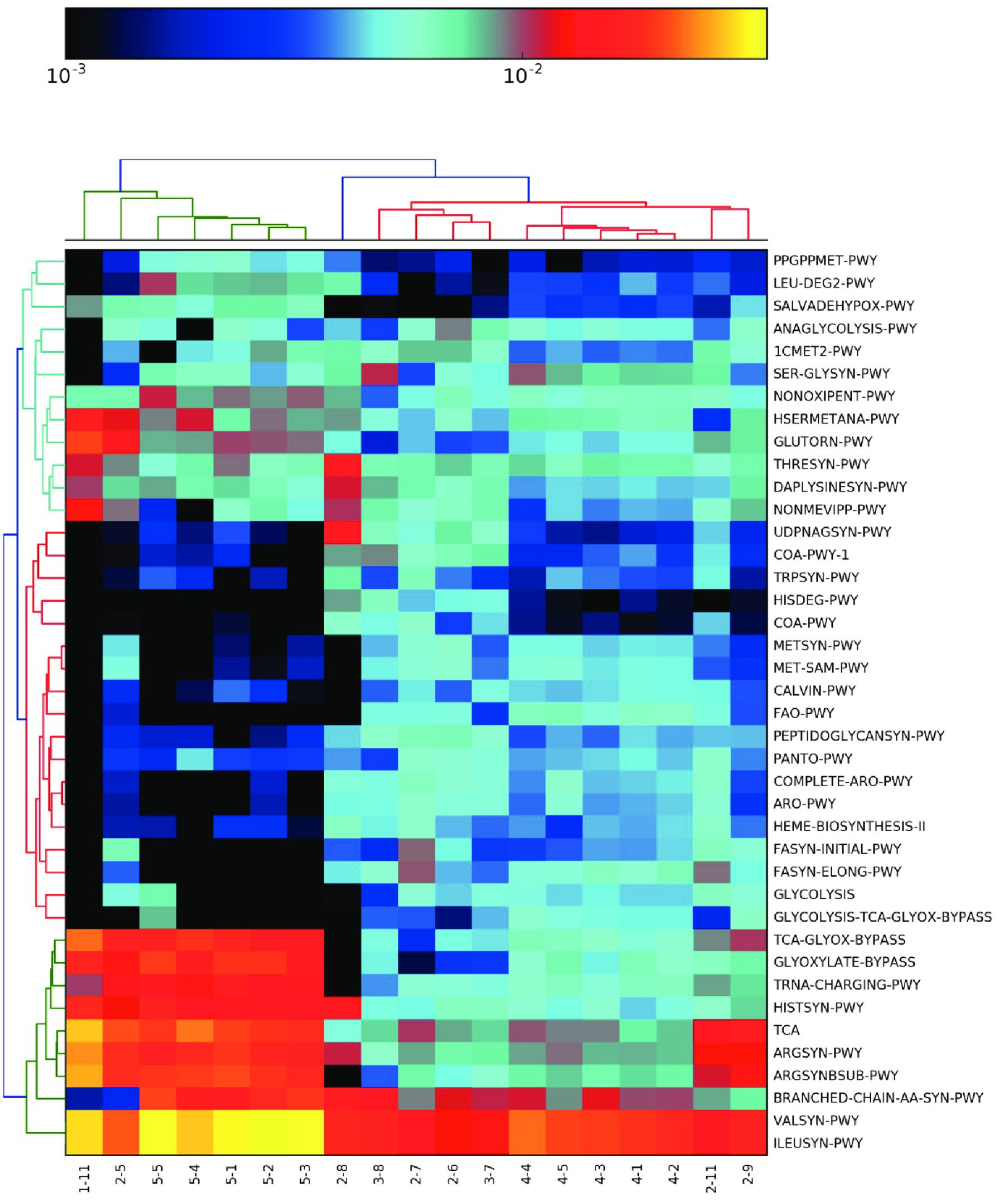
Hierarchical clustering of the top 40 pathways identified using HUMAnN2 for each sample (x-axis). See S11 Table in S2 File (https://zenodo.org/record/7041654) for full pathway names. Pathways from the whole metagenome sequence data for each sample were estimated using HUMAnN2. The results for each sample were merged into a single table. A heatmap with hierarchical clustering was generated for the top 40 pathways to provide a qualitative snapshot of grouping between samples. Complete pathway names are located in S11 Table in S2 File (https://zenodo.org/record/7041654).

A heatmap of relative abundances generated by HUMAnN2 for the 40 most abundant pathways in each sample is shown in Fig 8. The complete dataset is available in S11 Table in S2 File (https://zenodo.org/record/7041654). All samples had a high signal for the valine and isoleucine synthesis pathway and samples 1–11, 2–5 and all category 5 samples had a high abundance of tricarboxylic acid (TCA)-glyoxylate and glyoxylate bypass, TCA cycle, histidine and arginine synthesis and tRNA charging pathways. Category 5 also had a dominance of branched chain amino acid biosynthesis amino acid pathways. This suggests that these communities have the ability to grow in nutrient limited environments.

HUMAnN2 associates pathways with significant contributing organisms. These were plotted for the pathways mentioned above. For the L-arginine biosynthesis I pathway (ARGSYN) (S2 Fig https://zenodo.org/record/7041654), *Pseudomonas mendocina* was a contributor of the pathway in samples 2–5, 2–7 and 2–11, while *Staphylococcus epidermidis* was the only contributor in sample 2–6. *Enhydrobacter aerosaccus* was an important contributor in samples 3–7, 4–1, 4–2 and 4–3, yet much of the ARGSYN pathway was contributed by unclassified organisms in most samples, especially category 5, 2–5 and 1–11. The L-arginine biosynthesis II pathway (acetyl cycle; ARGSYNBSUB) was also mostly contributed by unclassified organisms (S3 Fig in S1 File https://zenodo.org/record/7041654), but *P*. *mendocina* was again present in 2–5, 2–7 and 2–11, by *E*. *aerosaccus* in category 4 vacuum debris samples and by *S*. *epidermidis* alone in sample 2–6. A somewhat different pattern was observed for the L-histidine biosynthesis pathway (HISTSYN) (S4 Fig in S1 File https://zenodo.org/record/7041654), where *P*. *mendocina* was again prevalent in 2–7 and 2–11 and also present in 2–5, but *Modestobacter multiseptatus* was a contributor of the pathway in 4–1, 4–2, 4–3 and 4–4, *C*. *acnes* in 2–8, 3–7 and 3–8, and *S*. *epidermidis* alone in 2–6. Many more organisms were assigned to the superpathway of branched amino acid biosynthesis (BRANCHED-CHAIN-AA-SYN) but again many were unclassified (S5 Fig in S1 File https://zenodo.org/record/7041654). Samples 2–5, 2–7 and 2–11 had major contributions by *P*. *mendocina*, 4–1, 4–2, 4–3 and 4–4 by *E*. *aerosaccus* with 4–3 and 4–4 including contributions from *Sphingobium yanoikuyae* and *Clavibacter michiganensis*. Category 5 samples were almost entirely unclassified. The TCA cycle I (prokaryotic) was largely contributed by two classes of unclassified organisms (dark blue and dark red in S6 Fig in S1 File https://zenodo.org/record/7041654). There was a contribution to the TCA cycle by *Massilia timonae*, especially in sample 2–9 and vacuum debris samples, a small contribution by *M*. *multiseptatus* in vacuum debris and a small contribution by *A*. *lwoffii* in category 5 samples. The glyoxylate cycle (GLYOXYLATE-BYPASS) (S7 Fig in S1 File https://zenodo.org/record/7041654) was completely contributed by unclassified organism in eleven samples, by *E*. *aerosaccus* and *M*. *multiseptatus* in category 4 samples and a very small contribution by *Kocuria rhizophila* (a persister species) in samples 3–7, 3–8 and 4–2. The superpathway of glyoxylate bypass and TCA (TCA-GLYOX-BYPASS) (S8 Fig in S1 File https://zenodo.org/record/7041654) and tRNA charging pathway (TRNA-CHARGING) (S9 Fig in S1 File https://zenodo.org/record/7041654) were unremarkable because nearly all the assignments were to unclassified organisms. The abundance of these organisms and their contributions to the pathways called agrees with the relative abundances of organisms predicted by both MetaPhlAn2 and Bracken (Figs 4 and 5; S7 and S8 Tables in S2 File https://zenodo.org/record/7041654).

A limitation of HUMAnN2 is that it uses the output from MetaPhlAn2. If MetaPhlAn2 cannot assign many of the reads because it is a marker-based database, then HUMAnN2’s output will also be limited. An alternative approach is to conduct read based annotation or assembly, as described below.

### KEGG-based functional analysis

The KEGG orthologs (Kos) with top 500 RPKM (Reads Per Kilobase per Million) values were used to estimate the functions with high copy count for each sample. The union of these functions identified a set of 2,704 KO numbers. The full table of these Kos is provided in S12 Table in S2 File (https://zenodo.org/record/7041654). We examined these KOs for the presence of functions related to spore formation, radiation resistance, anaerobiosis, peroxide resistance, *etc*. and found very few examples of proteins that have such functions. Only five proteins predicted for sporulation associated functions were annotated in a few of the samples. Most samples had one of four anaerobic reductases. All had catalase-peroxidase and organisms from category 4 samples had a nickel superoxide dismutase, yet no protein with annotated function associated with radiation resistance was observed.

**Fig 9:**
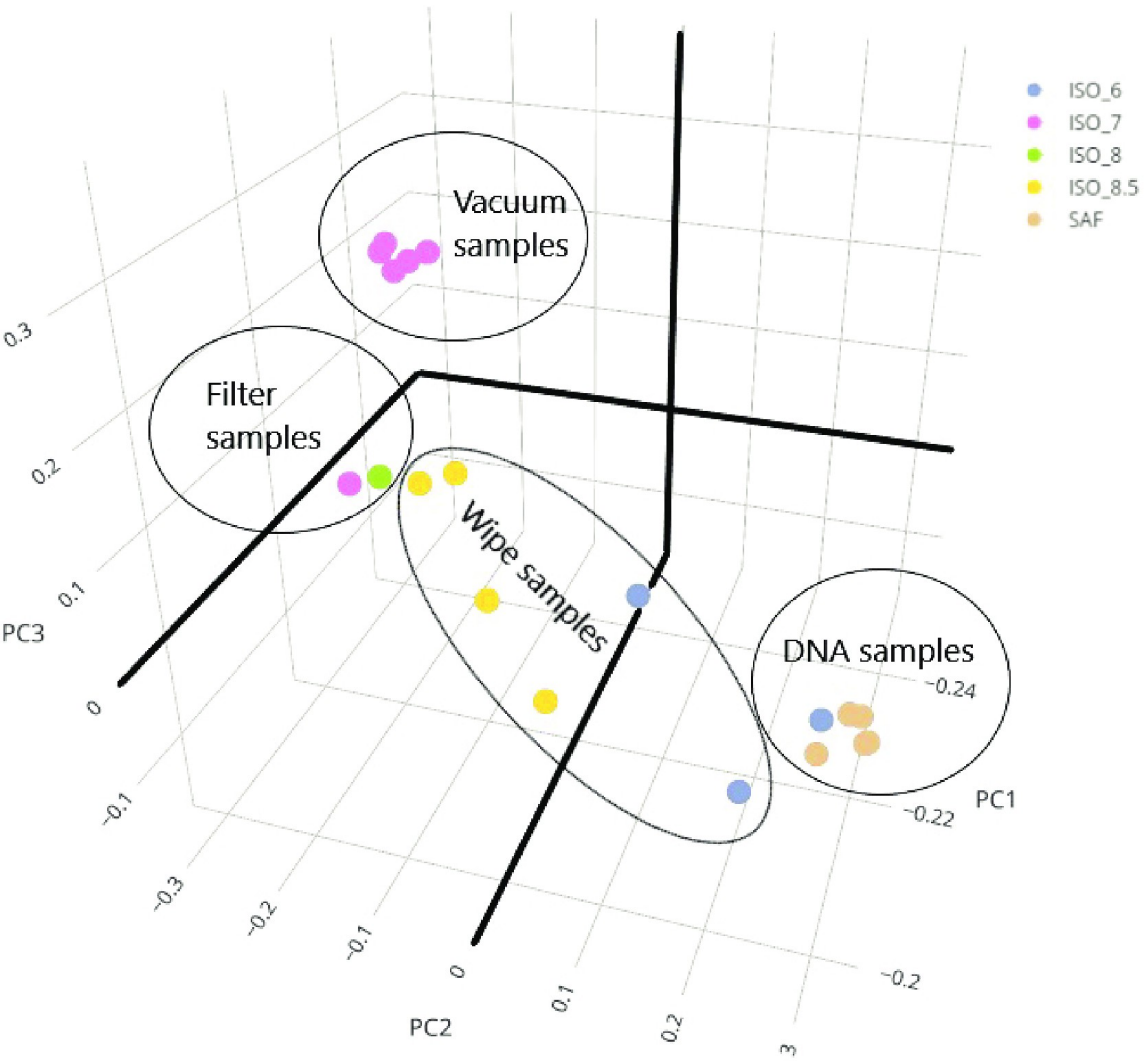
Sample clustering by KEGG function composition. A library of 6,741 KeGG Orthology (KO) functional sequences was collected for species and genera identified in the metagenomic analysis. Reads that uniquely mapped to a particular KO were counted and the counts were normalized for read depth and unique sequence length to generate RPKM-like statistics. The top 500 RPKM values were used to estimate the functions with high copy count for each sample. The union of these functions identified a set of 2,704 KOs. The figure shows the results of a principal components analysis (based on Bray-Curtis dissimilarity) across these KOs for all samples. Colors indicate ISO facilities and enclosing circles indicate different sample types. ISO represents building (or source), while circles identify sample types.

The Bray-Curtis dissimilarity between these KOs for all samples was used to identify the principal components (PCs) shown in Fig 9. Substantial clustering was observed on the basis of sample type and location. These results closely mirrored sample organization observed from taxonomic clustering of these samples. Sample 5–6 (not shown in Fig 9) was substantially different from all other samples. Of the top 500 functions drawn from each sample, 277 functions were unique to this sample. As mentioned above, this sample could not be classified using MetaPhlAN2 so it could not be analyzed using HUMANn2. This suggests a significantly different functional composition of the sample that is highly correlated to its taxonomic composition.

### Metagenome assembled genomes and annotation

We assembled sequence reads from the high-quality metagenome data sets using IDBA-UD [18], which is an assembler optimized for sequencing data that has highly uneven depth. Although the samples had very low microbial biomass, some were also low in diversity, so we expected that we might be able to capture near complete MAGs from these assembly efforts. In fact, we were able to generate MAGs from all (n = 18) but two of the metagenome data sets (S13 Table in S2 File https://zenodo.org/record/7041654). The assembly attempts for samples 4–3 and 5–6 never completed due to computational memory constraints, despite allocating 300 GB of memory to the job. The assembly-stats script was set to ignore contigs smaller than 1 kb. In many cases we were able to assemble contigs that were larger than 50 kb and in four cases the largest contig was larger than 150 kb.

### Taxonomic assignment of scaffolds

**Fig 10:**
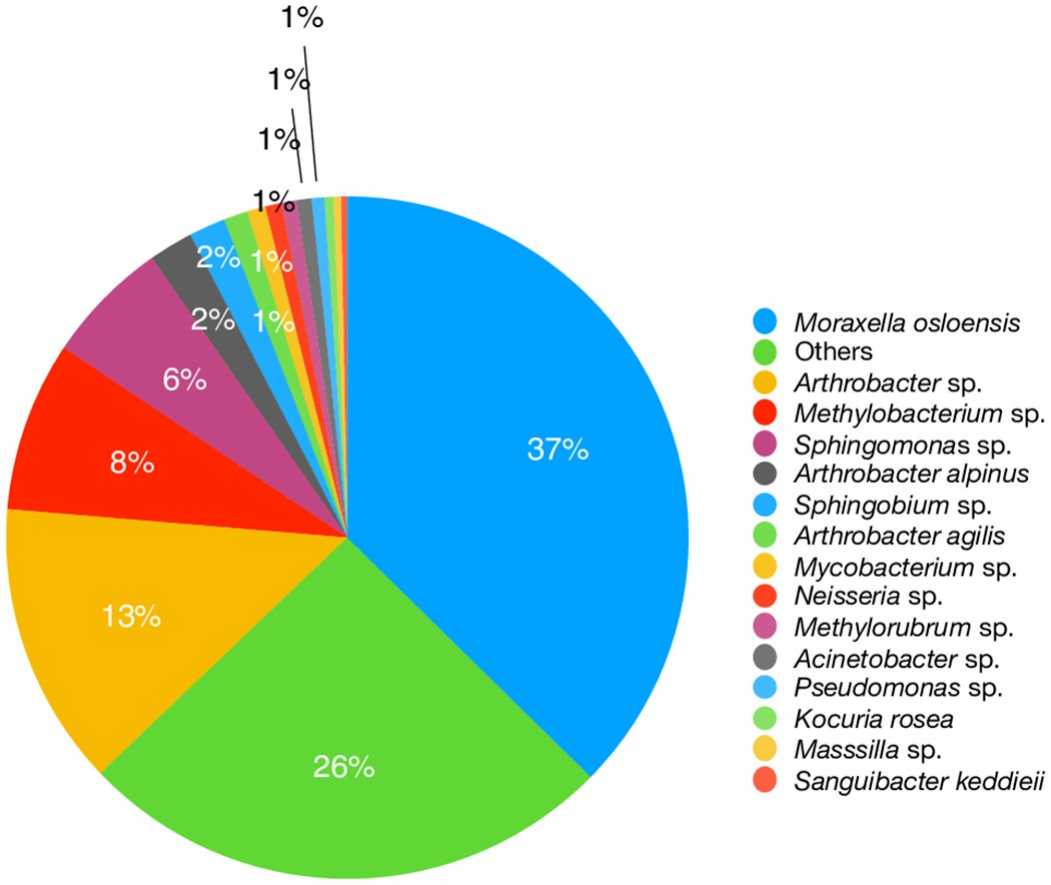
Taxonomy of the assembled metagenome of sample 4–1. Each contig greater than 1kb from the assembly of sample 4–1 was analyzed with blastn. The contribution of each organism was estimated. A pie chart was generated for the top hits to illustrate the taxonomic composition.

**Fig 11:**
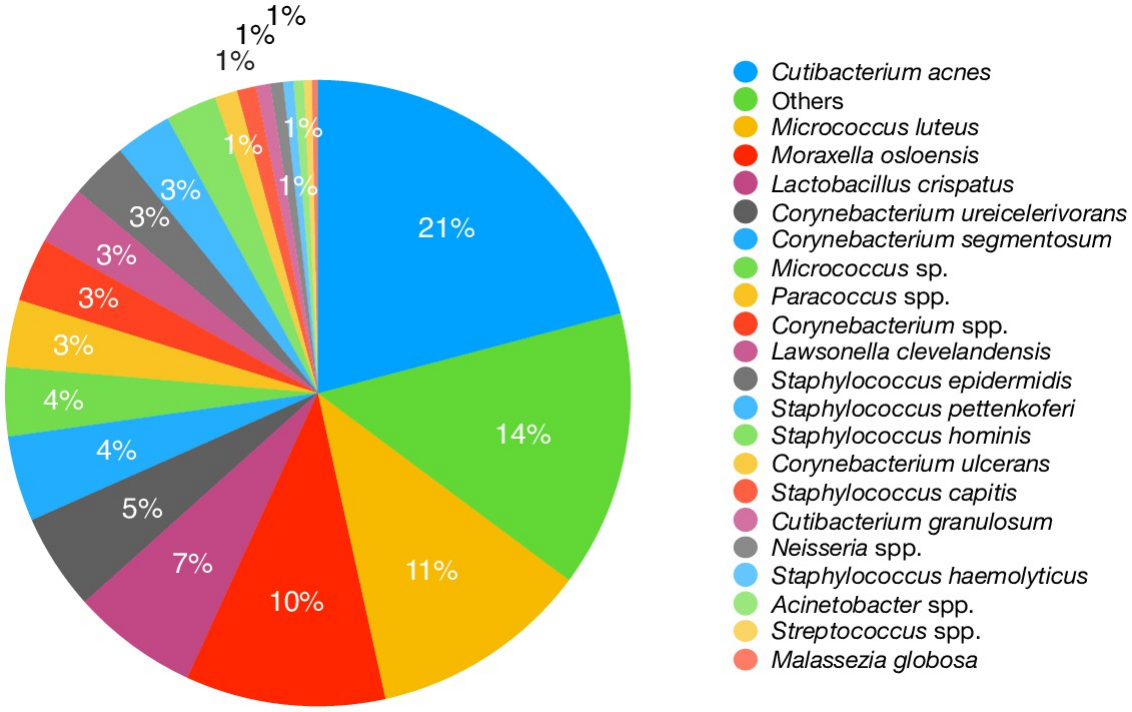
Taxonomy of the assembled metagenome of sample 3–7. Each contig greater than 1kb from the assembly of sample 3.7 was analyzed with blastn. The contribution of each organism was estimated. A pie chart was generated for the top hits to illustrate the taxonomic composition.

**Fig 12:**
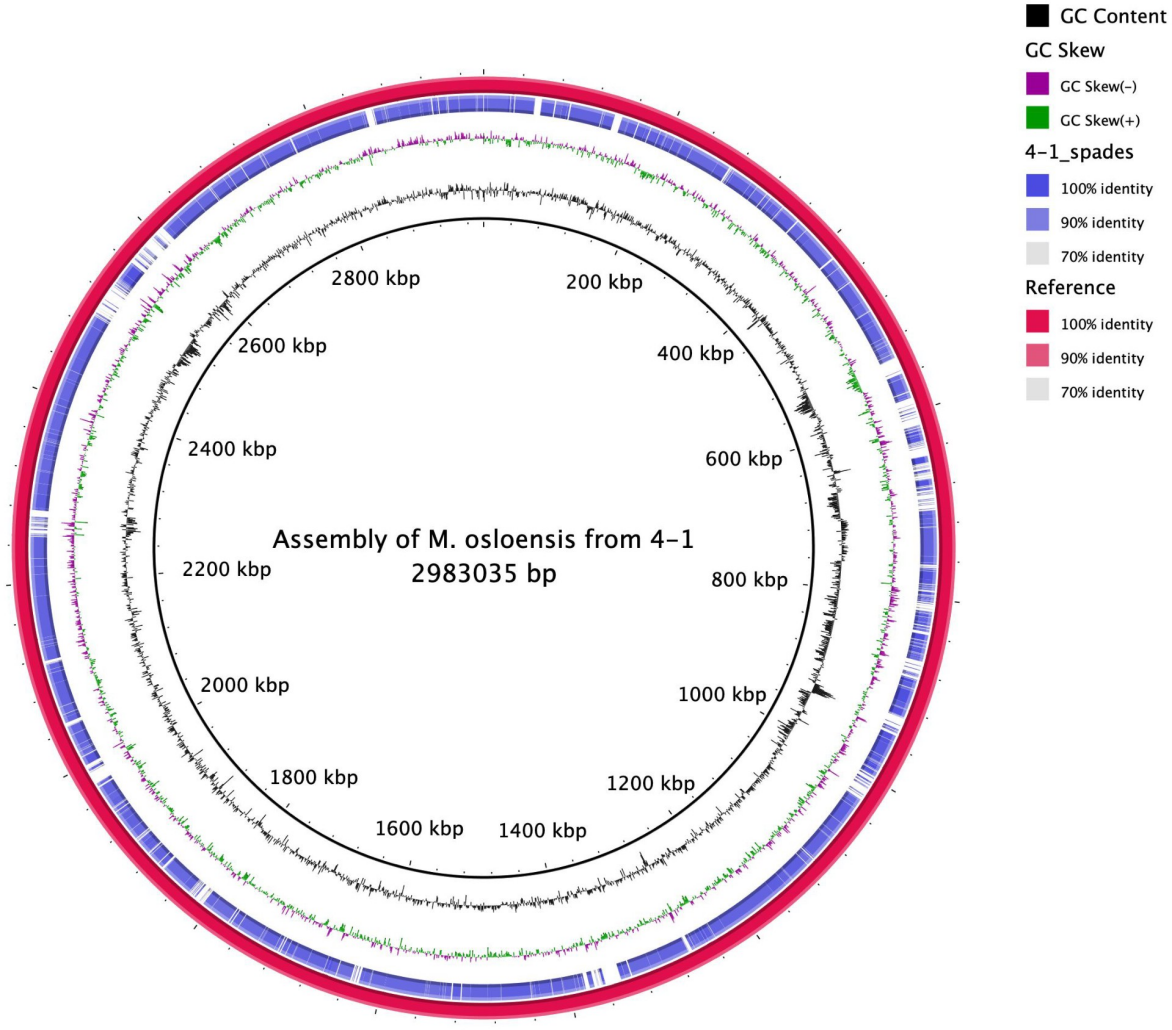
Circular map with open reading frames of the assembled genome of *Moraxella osloensis* from sample 4–1. Map was created with BLAST Ring Image Generator (BRIG) [28].

To conduct a detailed analysis on two assembled data sets, 4–1 (vacuum debris) and 3–7 (filter sample), blastn was run for each scaffold in the assembly that was ≥ 1 kb using the current version of GenBank to obtain the top hit for each contig (Table 5). We estimated the contribution of each organism in the assembly by summing the length of the alignments to each organism and dividing by the total number of bases in the assembly (see S14 and S15 Tables in S2 File https://zenodo.org/record/7041654 for contig blastn results and sums). A pie chart was generated for the top hits in samples 4–1 and 3–7 (Figs 10 and 11). In Fig 10, note that the most abundant species, *M*. *osloensis* (37%), is the same species identified as most abundant by Bracken (Fig 5) and MTSv (Fig 3). The assembly of sample 3–7 revealed a different distribution of species. *Malassezia restricta*, a skin fungus, was dominant (29%) and hits to all nine chromosomes were observed. It was only identified at the genus level by MTSv, but it was identified as *Malassezia globosa* by MetaPhlAn2 (S7 Table in S2 File https://zenodo.org/record/7041654). The second most abundant species, *C*. *acnes* (15%) was identified by all three read-based methods. *M*. *osloensis* (8%) was also identified in this sample. The *M*. *osloensis* reads from sample 4–1 were assembled from the metagenome by taxonomically binning the reads and running a whole genome assembly assembled using SPAdes [19] (Fig 12). The genome was also annotated using Prokka (S16 Table in S2 File https://zenodo.org/record/7041654). The annotation provided 1992 predicted open reading frames, four ribosomal RNA genes, three of which were partial, and 41 tRNA genes. Although classical typing characterized *M*. *osloensis* to be non-motile [25], we identified proteins associated with the production of type IV pilus, which is common in other *Moraxella* species [26]. Several chemotaxis proteins (CheB, CheW, and CheZ) were also identified. This reference-free MAG generated a single circular scaffold of ~2.89 mbp, which is remarkably close to the published ~2.5 mbp chromosome sizes from cultured *M*. *osleonsis* [27]. In addition to the larger assemblies, scaffolds assembled from each data set allowed the identification of their closest taxonomic relative from the NCBI nt database. This was highly successful with high quality identities for 75%, or more, of the scaffolds across the study (S17 Tbale in S3 File and S18 Table in S3 File https://zenodo.org/record/7041654). In all, over 800,000 taxonomic identities were assigned to scaffolds.

**Table 5:** Scaffold taxonomic identity.

Sample	Total scaffolds	Blast annotated Scaffolds	Scaffolds w/o BLAST hits	Hiqh-Quality Scaffolds	Scaffolds w/o high-quality hits
2–5	32,110	22,736	29%	18,094	20.4%
2–6	37,821	22,964	39%	20,770	9.6%
2–7	39,289	33,080	16%	28,957	12.5%
2–8	11,752	8,152	31%	7,476	8.3%
2–9	311,761	230,584	26%	197,269	14.4%
3–7	240,759	179,422	25%	161,424	10.0%
3–8	29,769	25,566	14%	23,534	7.9%
4–1	177,234	84,909	52%	61,242	27.9%
4–2	53,719	29,695	45%	19,506	34.3%
4–4	112,276	69,292	38%	53,092	23.4%
4–5	20,230	7,438	63%	5,329	28.4%
5–1	12,190	9,465	22%	7,382	22.0%
5–2	5,039	4,027	20%	2,986	25.9%
5–3	14,970	11,569	23%	8,898	23.1%
5–4	11,921	9,349	22%	7,630	18.4%
5–5	10,210	7,949	22%	6,252	21.3%
1–11	7,812	5,944	24%	4,519	24.0%
2–11	75,329	55,148	27%	44,985	18.4%

## Discussion

Environmental metagenomic characterization of the spacecraft assembly facilities is critical to understand their potential microbial diversity and will lead to better risk assessment and improve methods to reduce the risk of accidental interplanetary contamination. Using whole metagenome sequencing and multi-classification bioinformatic analysis, we generated high confidence identification of microorganisms associated with floor wipe and filter samples at the JPL SAF. The 16S rRNA and 18S rRNA counts are generally correlated with ISO classification, with the SAF (Blg 179) having the highest counts of bacteria and fungi. Use of three different classification methods provided a comprehensive classification of samples. Not surprisingly, dominantly identified organisms were mostly those associated with human skin, such as *C*. *acnes*, *A*. *lwoffii* and *Malassezia*, or were environmental organisms in the phyla Actinobacteria and Proteobacteria. Category 4 samples (vacuum particles from cleanroom vacuums) were of most concern to Planetary Protection because they included a number of taxa with the potential to be resistant to desiccation, radiation, UV irradiation, low temperature, heavy metals, *etc*. Many of these and others were oligotrophs that can survive under nutrient limiting conditions. These included members of the Geodermatophilaceae (*Blastococcus*, *Geodermatophilus* and *Modestobacter*), *Methylobacterium*, *Deinococcus*, sphingomonads, and *Stenotrophomonas*. We identified 17 (12 in top 40%) of 22 genera recently described as being newly isolated from the International Space Station [29], suggesting that our methods were comprehensive.

Functional annotation failed to identify abundant protein families associated with spore formation, desiccation resistance, radiation resistance or other properties that would allow organisms to survive in extreme environments. Nevertheless, our HUMANn2 analysis of pathways and organisms provided a clue. One of the key mechanisms of radiation and other defiance in *D*. *radiodurans* is resistance to reactive oxygen species (ROS) [30]. Catalase and superoxide dismutase can contribute to ROS resistance, but another significant mechanism is *via* the glyoxylate bypass of the TCA cycle [31,32], which avoids the free radical generating steps of the TCA cycle. In the HUMANnN2 analysis, the glyoxylate bypass of the TCA cycle was only associated with *Modestobacter multiseptatus* in vacuum debris samples, yet the glyoxylate cycle was associated with additional organisms in the ULPA filter and 4 vacuum debris samples.

Our analyses were limited by low biomass in samples, as such, some could not be submitted to WGS. Focused DNA sequencing strategies, such as PCR based amplicons, can provide a superior limit of detection (LOD) while still generating genomic sequences to enhance sensitivity and specificity in the analysis. In contrast, WMGS, as performed in this study, provided the opportunity to query samples in an unbiased fashion to identify unanticipated environmental components. Clearly, both are valuable approaches with particular attributes that provide complementary information. Focused sequencing amplicons can be developed for specific microbial targets from comparative genomic analysis. This would provide for the appropriate sensitivity and specificity, while gaining the superior LOD. When there are known high consequence microbes (examples of which are reported here) that must be detected, a focused amplicon approach should be developed and included in an ongoing monitoring program. Future decreases in cost per read, increases in sequencing fidelity, and longer read lengths will make MWGS even more powerful.

## Conclusions

As humanity embarks on interplanetary exploration and travel, we must continue to improve our methods to reduce chances of space contamination with earth-based microbes. Recent advances in metagenomic sequencing and bioinformatic analyses is making this possible. Here we gained valuable insight on both microbial burden in spacecraft and instrument manufacturing facilities but also in appropriate analysis methodologies. Classification of metagenomic reads is both algorithm and database dependent, and individually these methods provide unique but incomplete views of the environmental microbiome, even in a highly controlled, low microbial burden setting as the JPL facilities described here. Using a multi-classification approach, we believe that we have captured a majority of the known microbial taxa within the JPL cleanroom environments. WMGS is clearly a powerful technique for unbiased monitoring of microbial burden of spacecraft assembly facilities, both in terms of presence and likely functional impact. Assembled WMGS data can also be used to capture new microbial “dark matter”, microbes that lack current database representation and, as such, may represent previously unforeseen challenges to space exploration. A comprehensive, mulit-classification metagenomic approach is therefore critical for environmental monitoring of ongoing and future spacecraft and instrument manufacturing.

## Supporting information

S1 File S1-S9 Figs https://zenodo.org/record/7041654). (PDF)

S2 File S1-16 Tables (https://zenodo.org/record/7041654). (XLSX)

S3 File S17—Assemblies Genus Identity Table (https://zenodo.org/record/7041654). (XLSX)

S4 File S18—Assemblies Species Identity Table (https://zenodo.org/record/7041654). (XLSX)
