# Supplementary material for: Multi-faceted metagenomic analysis of spacecraft associated surfaces reveal planetary protection relevant microbial composition
Source: PLoS One. 2023 Mar 22;18(3):e0282428. doi: 10.1371/journal.pone.0282428 (PMC10032485; doi:10.1371/journal.pone.0282428)
Supplement: S1 File — (PDF) [file pone.0282428.s001.pdf]

Supplemental Figures for:

Multi-faceted metagenomic analysis of spacecraft associated surfaces reveal planetary protection relevant microbial composition

Sarah K. Highlander [1,ŧ] Jason M. Wood [2], John D. Gillece [1,3], Megan Folkerts [1], Viacheslav Fofanov [3,4], Tara Furstenau [3], Nitin K. Singh [2], Lisa Guan [2], Arman Seuylemezin [2], James N. Benardini [2], David M. Engelthaler[1], Kasthuri Venkateswaran [2,*], and Paul S. Keim[1,3,*]

[1] Pathogen and Microbiome Division, The Translational Genomics Research Institute, 3051 W. Shamrell Blvd, Suite 106, Flagstaff, AZ

[2] Biotechnology and Planetary Protection Group, Jet Propulsion Laboratory, California Institute of Technology, Pasadena, CA

[3] Pathogen & Microbiome Institute (PMI), Northern Arizona University, Flagstaff, AZ

[4] School of Informatics, Computing and Cyber Systems, Northern Arizona University, Flagstaff, AZ

*** Correspondence:**

Dr. Paul Keim

Pathogen and Microbiome Institute

Northern Arizona University

Flagstaff, AZ 86011-4073

Tel: (928) 523-1078; Fax: (928)-523-4015
E-mail: Paul.Keim@nau.edu

Dr. Kasthuri Venkateswaran (Venkat)
Senior Research Scientist
California Institute of Technology, Jet Propulsion Laboratory
Biotechnology and Planetary Protection Group; M/S 245-104
4800 Oak Grove Dr., Pasadena, CA 91109
Tel: (818) 393-1481; Fax: (818) 3934176
E-mail: kjvenkat@jpl.nasa.gov

ǂDeceased.

Current addresses: JMW: University of Illinois at Chicago, 1200 W Harrison St, Chicago, IL 60607. JNB: NASA, 300 E St SW, Washington, DC 20546

ABSTRACT The National Aeronautics and Space Administration (NASA) has been monitoring the microbial burden of spacecraft since the 1970's Viking missions. Originally culture-based and then focused 16S sequencing techniques were used, but we have now applied whole metagenomic sequencing to a variety of cleanroom samples at the Jet Propulsion Lab (JPL), including the Spacecraft Assembly Facility (SAF) with the goals of taxonomic identification and for functional assignment. Our samples included facility pre-filters, cleanroom vacuum debris, and surface wipes. The taxonomic composition was carried out by three different analysis tools. Hierarchical clustering analysis separated vacuum particles from SAF DNA samples. Vacuum particle samples were the most diverse while DNA samples from the ISO facilities and the SAF were the least diverse; all three were dominated by Proteobacteria. Wipe samples had higher diversity and were predominated by Actinobacteria, including human commensals *Cutibacterium acnes* and *Corynebacterium* spp. Taxa identified by the three methods were not identical, supporting the use of multiple methods for metagenome characterization. Likewise, functional annotation was performed using multiple methods. Vacuum particle and SAF tricarboxylic acid cycle and amino acid biosynthesis suggested that many of the identified microorganisms have the ability to grow in nutrient-limited environments. In total, 18 high quality metagenome assembled genomes (MAG) were generated from the metagenomic sequences (MAG) and were dominated by *Moraxella osloensis* or *Malassezia restricta*. A *M. osloensis* MAG was assembled into a single circular scaffold and gene annotated. This includes a rigorous quantitative determination of microbial loads, and a qualitative dissection of microbial composition. Assembly of multiple specimens led to greater confidence for the identification of particular species and their predicted functional roles.

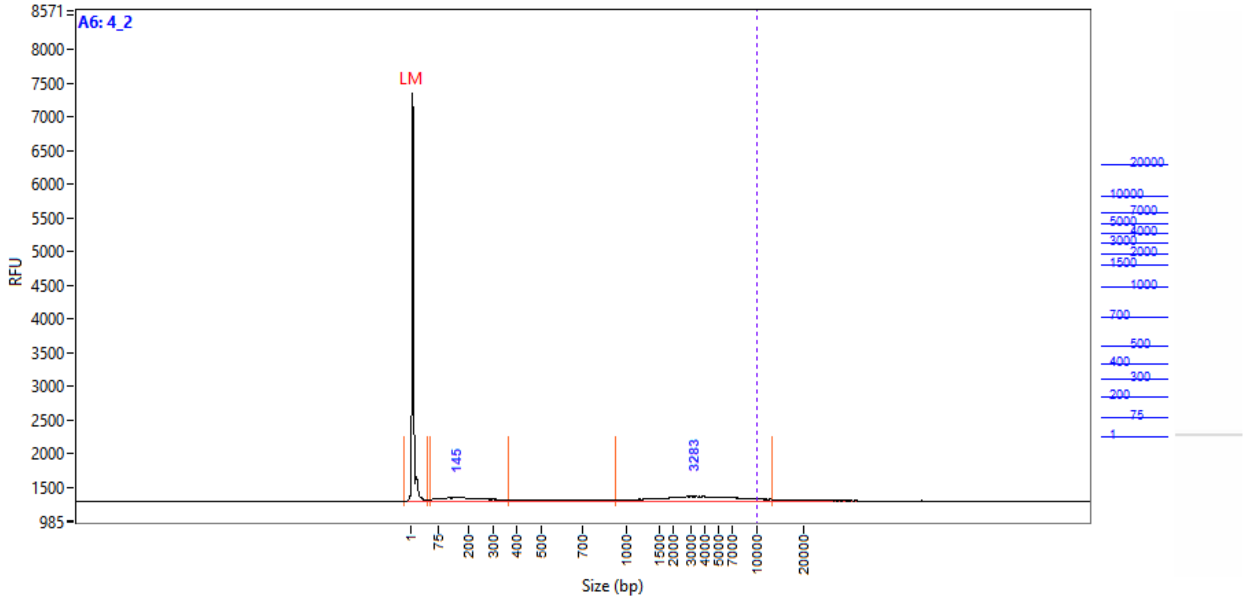

Supplemental Figure 1a: Example of a Fragment Analyzer electropherogram of a sample with sufficient total DNA. Sample shown is 4-2, which had a BactQuant yield of ~85,000 16S rRNA gene copies/µL. Note the genomic DNA peak at 3200 bp. The total estimated DNA concentration for this sample was 217 picograms/µL.

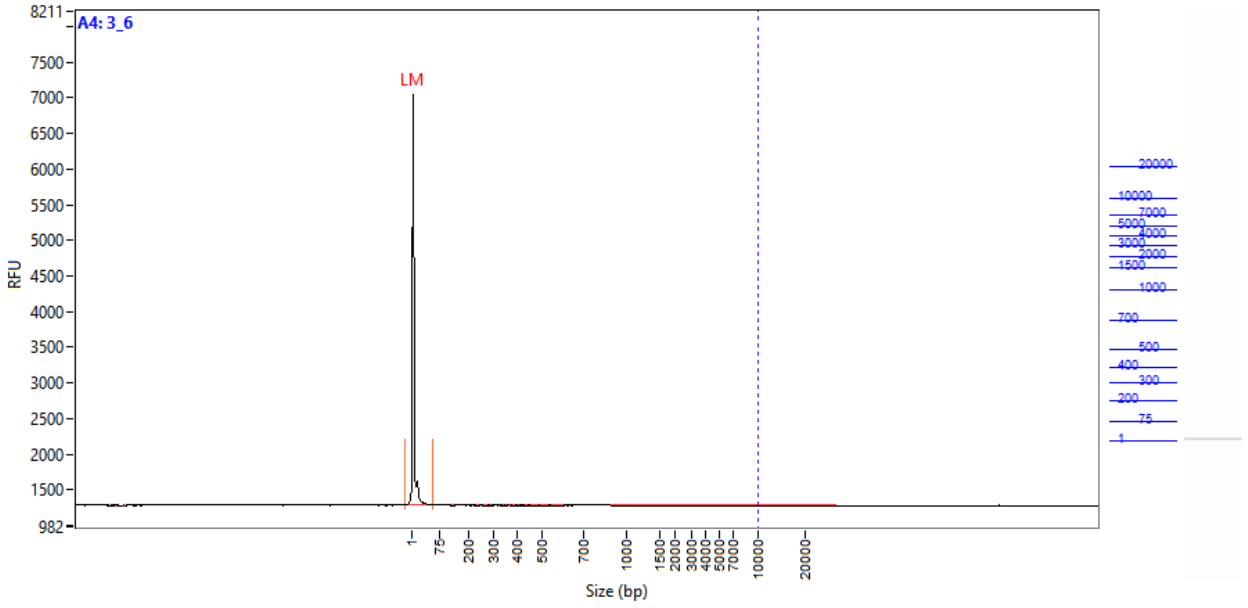

Supplemental Figure 1b: Example of a Fragment Analyzer electropherogram of a sample with no measurable DNA. Sample shown is 3-6, which was near the detection limit for both BactQuant (307 16S copies/µL) and FungiQuant (10 copies/µL). With the exception of the lower marker (LM), no other DNA peaks are visible. Fragment analysis estimated the concentration of this sample to be below the detection limit of 5 picograms/µL.

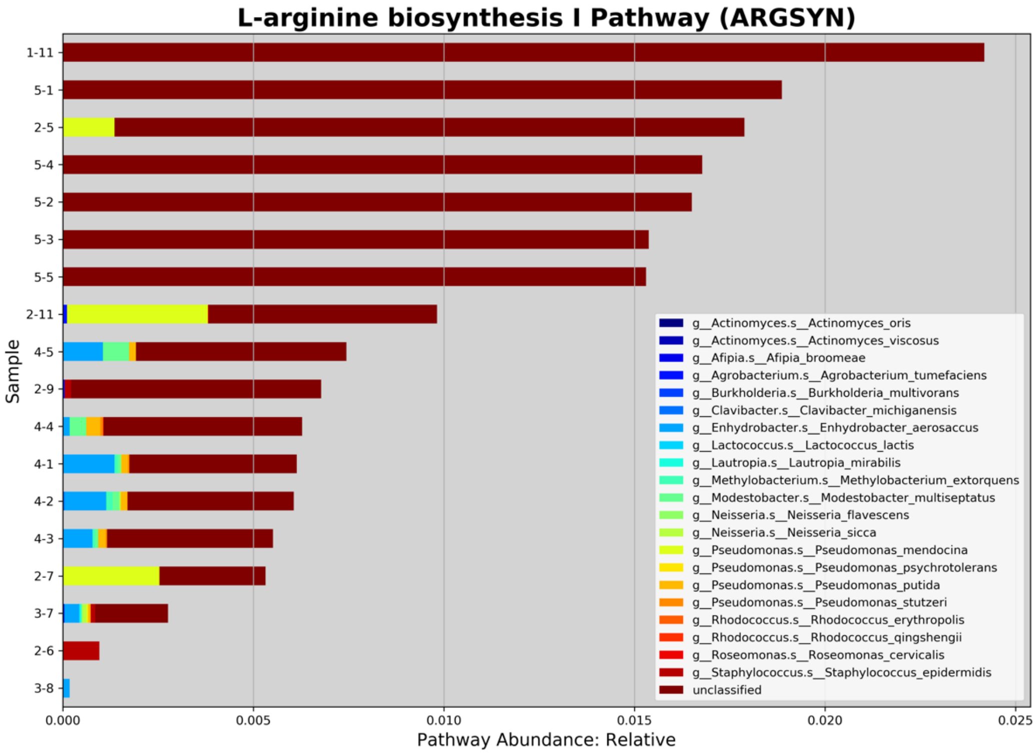

Supplemental Figure 2: Relative abundance of L-arginine biosynthesis I pathway by organism in samples from high quality libraries (y-axis). The bar chart generated using HUMANn2 illustrates the relative abundance of the L-arginine biosynthesis I pathway as well as from which species it was likely derived.

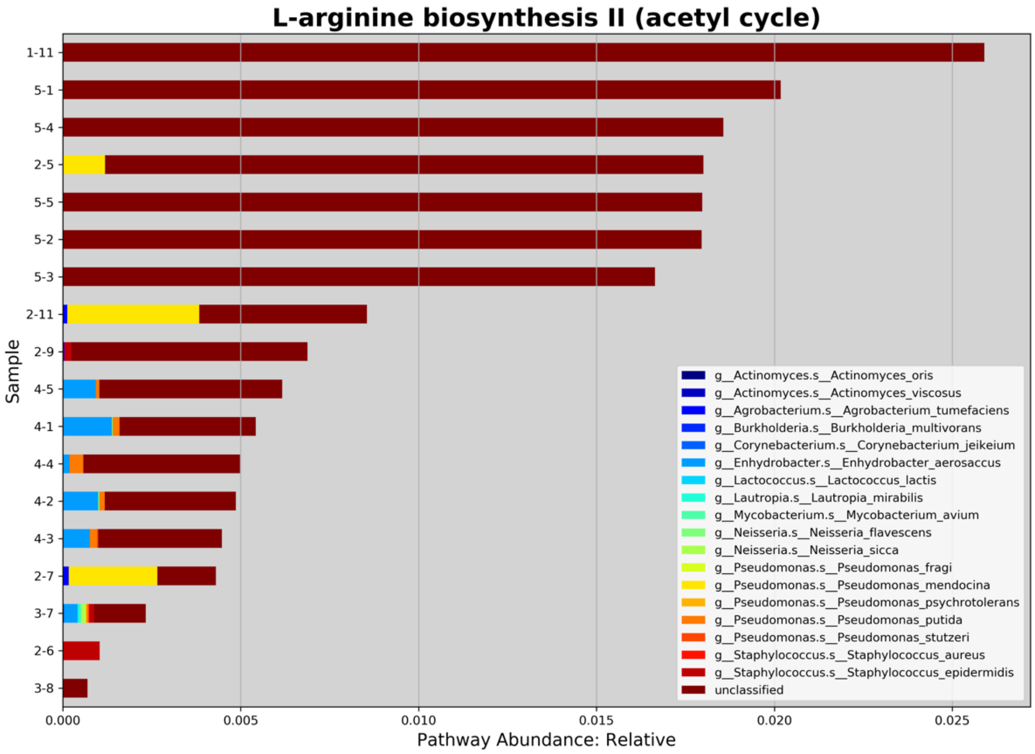

Supplemental Figure 3: Relative abundance of L-arginine biosynthesis II pathway by organism in samples from high quality libraries (y-axis).

Supplemental Figure 3: The bar chart generated using HUMANn2 illustrates the relative abundance of the L-arginine biosynthesis II pathway as well as from which species it was likely derived.

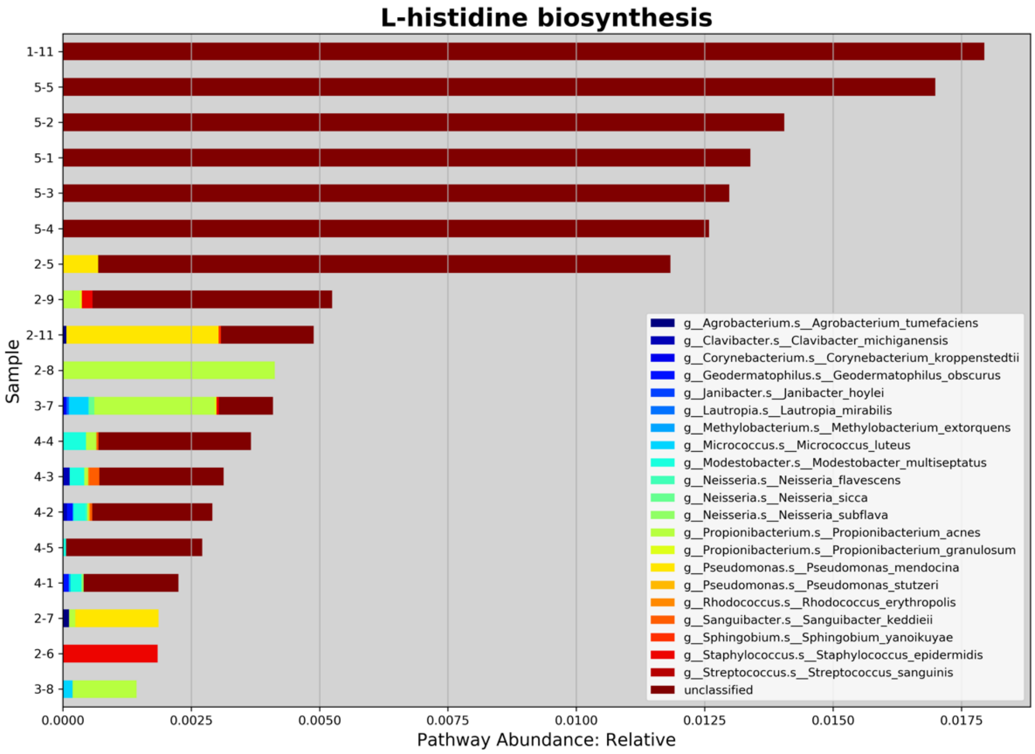

Supplemental Figure 4: Relative abundance of L-histidine biosynthesis pathway by organism in samples from high quality libraries (y-axis). The bar chart generated using HUMANn2 illustrates the relative abundance of the L-histidine biosynthesis pathway as well as from which species it was likely derived.

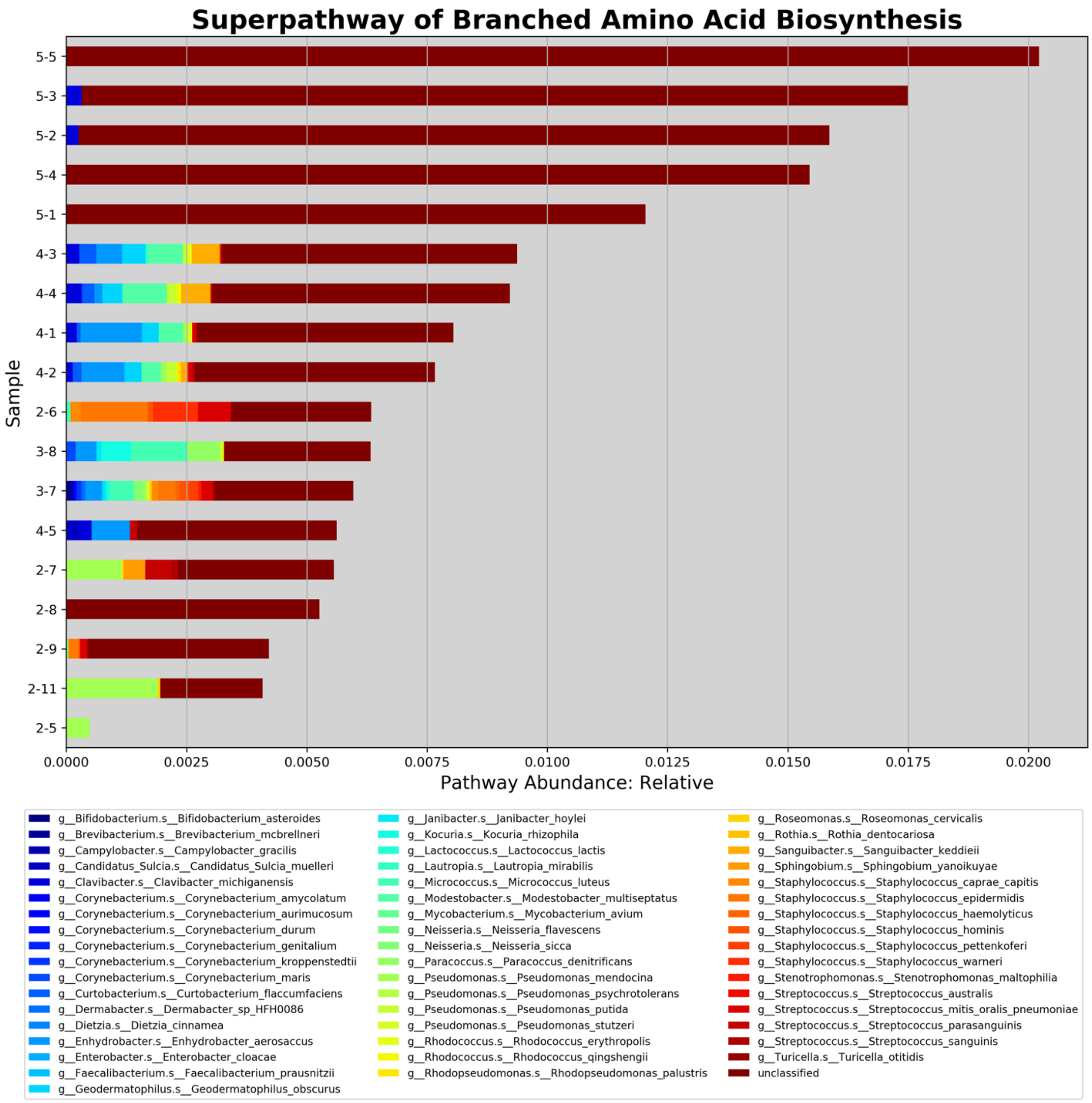

Supplemental Figure 5: Relative abundance of branched amino acid biosynthesis pathway by organism in samples from high quality libraries (y-axis). The bar chart generated using HUMANn2 illustrates the relative abundance of the branched amino acid biosynthesis pathway as well as from which species it was likely derived.

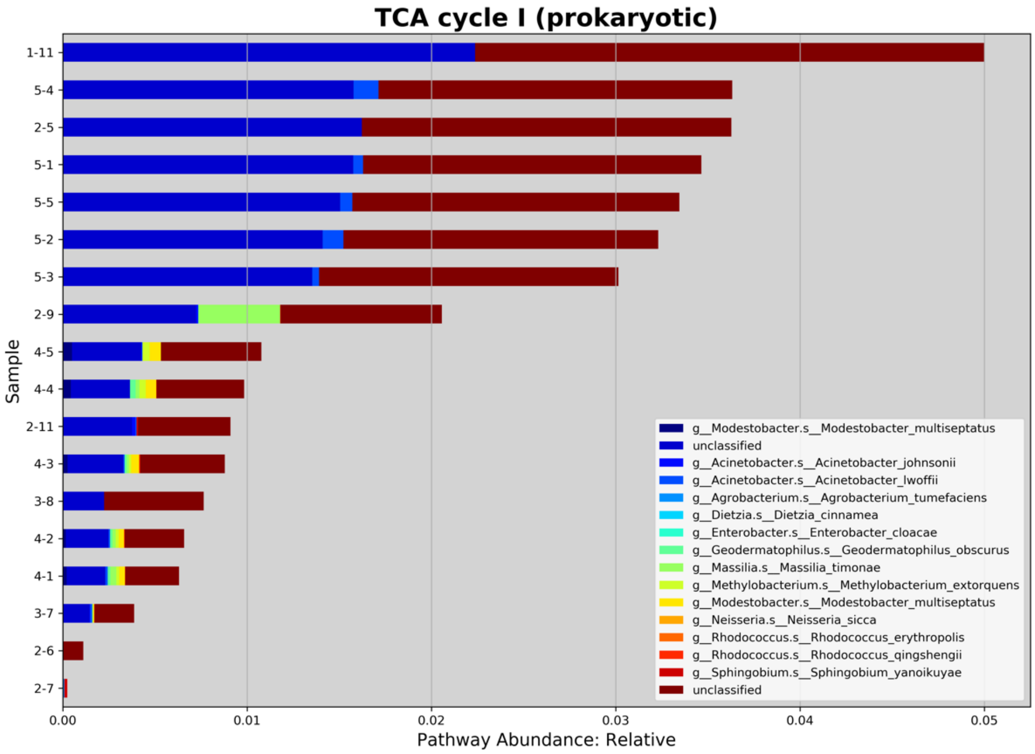

Supplemental Figure 6: Relative abundance of TCA cycle I pathway by organism in samples from high quality libraries (y-axis). The bar chart generated using HUMANn2 illustrates the relative abundance of the TCA cycle I pathway as well as from which species it was likely derived.

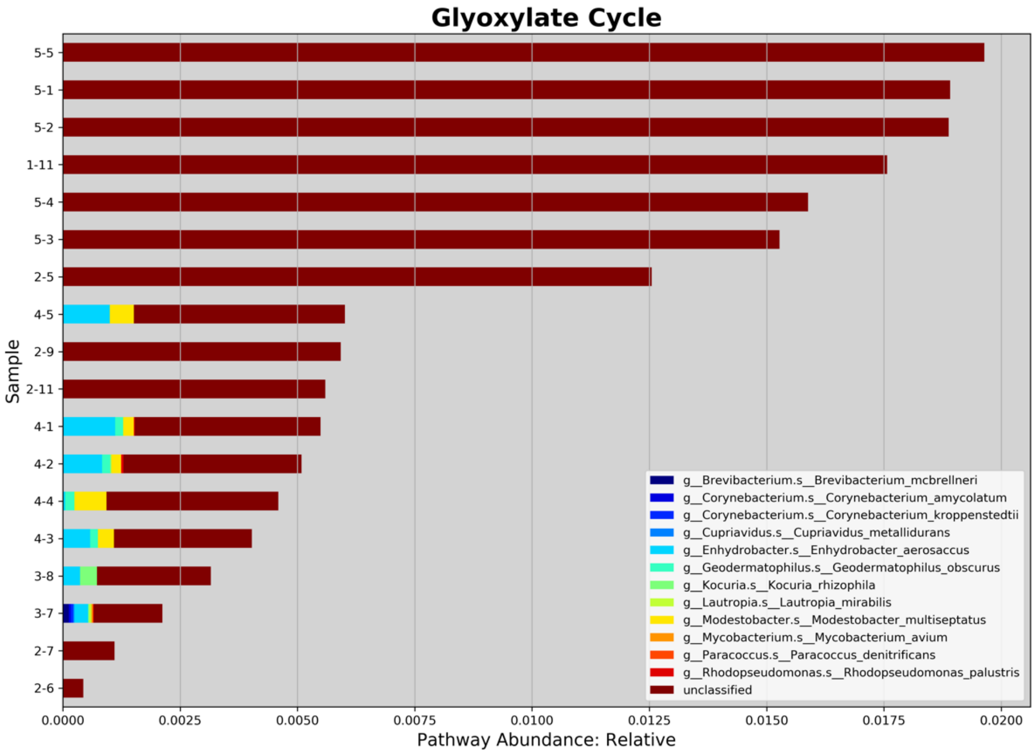

Supplemental Figure 7: Relative abundance of glyoxylate cycle pathway by organism in samples from high quality libraries (y-axis). The bar chart generated using HUMANn2 illustrates the relative abundance of the glyoxylate cycle pathway as well as from which species it was likely derived.

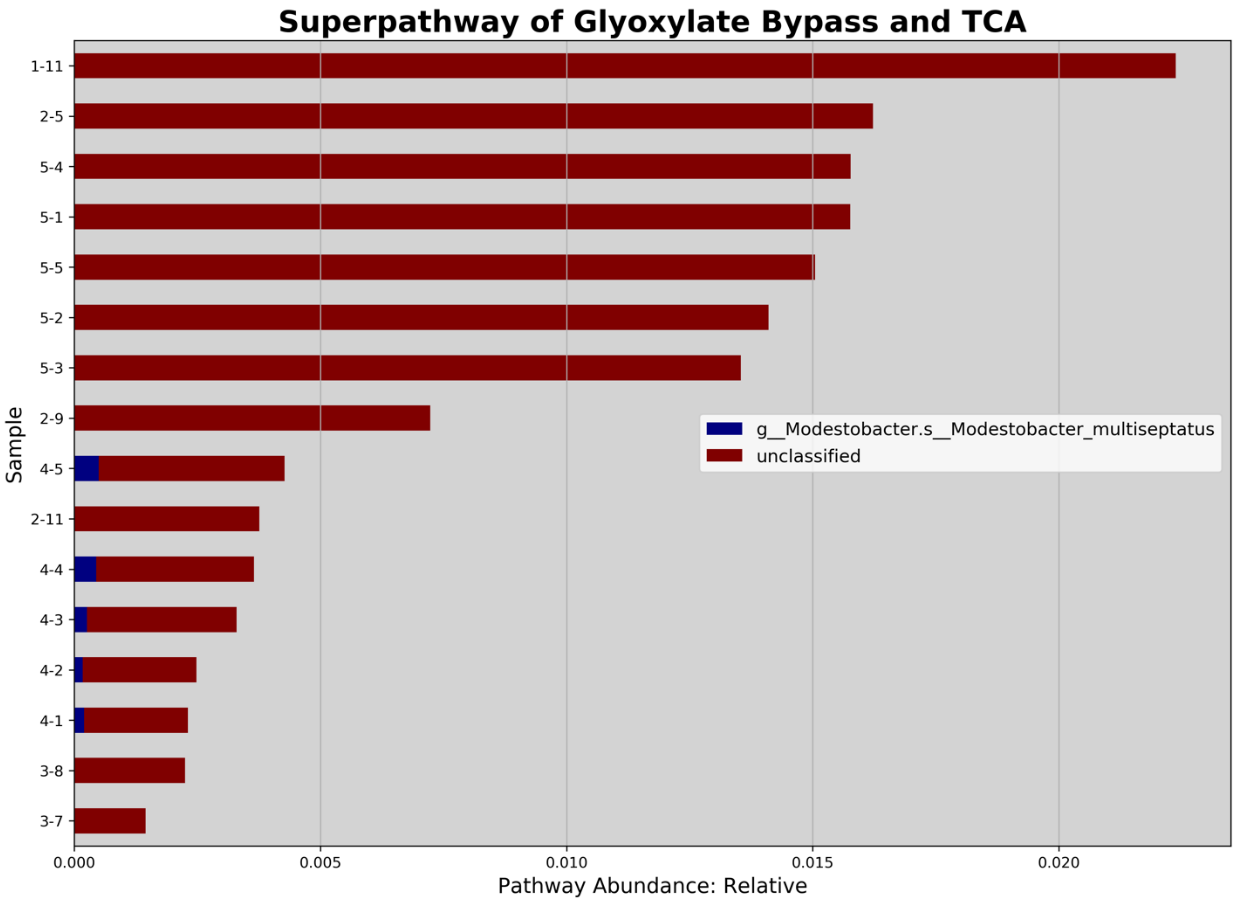

Supplemental Figure 8: Relative abundance of glyoxylate bypass and TCA cycle superpathway by organism in samples from high quality libraries (y-axis). The bar chart generated using HUMANn2 illustrates the relative abundance of the glyoxylate bypass and TCA cycle super pathway as well as from which species it was likely derived.

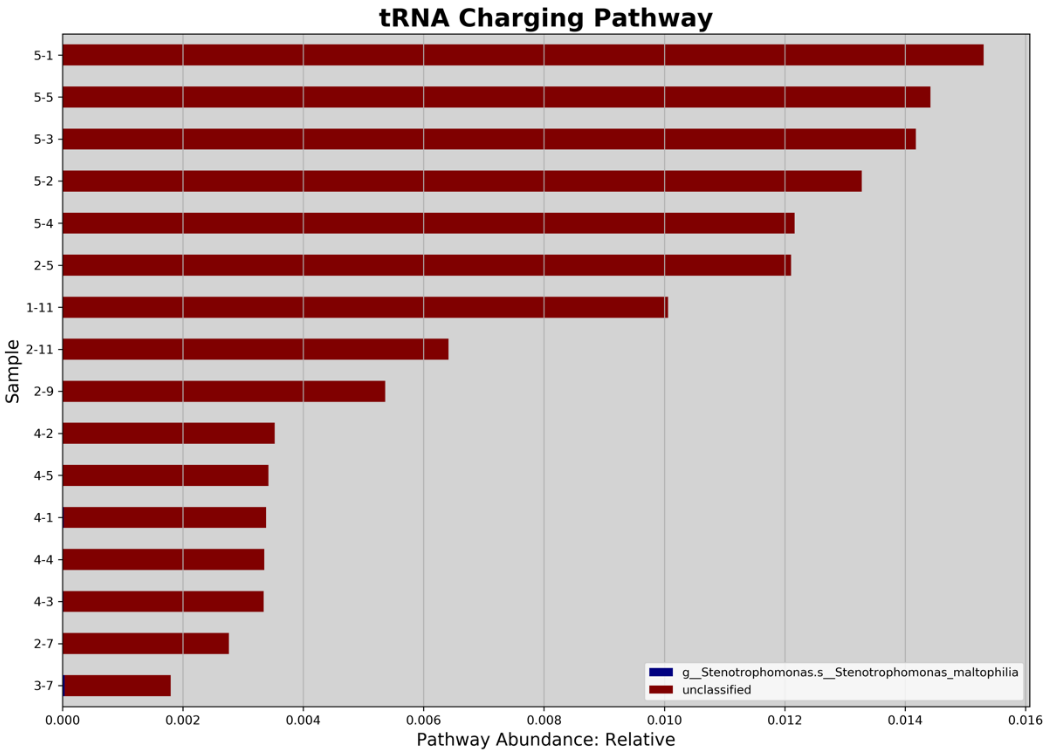

Supplemental Figure 9: Relative abundance of tRNA charging pathway by organism in samples from high quality libraries (y-axis). The barchart generated using HUMANn2 illustrates the relative abundance of the tRNA charging pathway as well as from which species it was likely derived.
